# Characterization of Alzheimer’s tau biomarker discordance using plasma, CSF, and PET

**DOI:** 10.1186/s13195-021-00834-3

**Published:** 2021-05-04

**Authors:** Yu Guo, Yu-Yuan Huang, Xue-Ning Shen, Shi-Dong Chen, Hao Hu, Zuo-Teng Wang, Lan Tan, Jin-Tai Yu, the Alzheimer’s Disease Neuroimaging Initiative

**Affiliations:** 1 Department of Neurology and Institute of Neurology, Huashan Hospital, Shanghai Medical College, Fudan University, Shanghai, China; 2 Department of Neurology, Qingdao Municipal Hospital, Qingdao University, Qingdao, China; 3 Department of Neurology, Qingdao Municipal Hospital, College of Medicine and Pharmaceutics, Ocean University of China, Qingdao, China

**Keywords:** Alzheimer’s disease, Plasma p-tau181, CSF p-tau181, AV1451 PET, Amyloid-β

## Abstract

**Background:**

We aimed to investigate the tau biomarker discrepancies of Alzheimer’s disease (AD) using plasma tau phosphorylated at threonine 181 (p-tau181), cerebrospinal fluid (CSF) p-tau181, and AV1451 positron emission tomography (PET).

**Methods:**

In the Alzheimer’s Disease Neuroimaging Initiative, 724 non-demented participants were categorized into plasma/CSF and plasma/PET groups. Demographic and clinical variables, amyloid-β (Aβ) burden, flortaucipir-PET binding in Braak regions of interest (ROIs), longitudinal changes in clinical outcomes, and conversion risk were compared.

**Results:**

Across different tau biomarker groups, the proportion of participants with a discordant profile varied (plasma+/CSF− 15.6%, plasma−/CSF+ 15.3%, plasma+/PET− 22.4%, and plasma−/PET+ 6.1%). Within the plasma/CSF categories, we found an increase from concordant-negative to discordant to concordant-positive in the frequency of Aβ pathology or cognitive impairment, rates of cognitive decline, and risk of cognitive conversion. However, the two discordant categories (plasma+/CSF− and plasma−/CSF+) showed comparable performances, resulting in similarly reduced cognitive capacities. Regarding plasma/PET categories, as expected, PET-positive individuals had increased Aβ burden, elevated flortaucipir retention in Braak ROIs, and accelerated cognitive deterioration than concordant-negative persons. Noteworthy, discordant participants with normal PET exhibited reduced flortaucipir uptake in Braak stage ROIs and slower rates of cognitive decline, relative to those PET-positive. Therefore, individuals with PET abnormality appeared to have advanced tau pathological changes and poorer cognitive function, regardless of the plasma status. Furthermore, these results were found only in individuals with Aβ pathology.

**Conclusions:**

Our results indicate that plasma and CSF p-tau181 abnormalities associated with amyloidosis occur simultaneously in the progression of AD pathogenesis and related cognitive decline, before tau-PET turns positive.

**Supplementary Information:**

The online version contains supplementary material available at 10.1186/s13195-021-00834-3.

## Background

Alzheimer’s disease (AD) has a decades-long period of pathologic alterations before dementia onset [1, 2]. This provides the opportunity to delay disease occurrence or even prevent AD dementia by intervening in the preclinical stage [3, 4]. Such early interventions require supportive approaches to promptly identify individuals at high risk of developing AD [3]. As one of the pathological hallmarks of AD, tau pathology can be detected by plasma, cerebrospinal fluid (CSF), and positron emission tomography (PET) assays [5]. It is typically unavailable to evaluate the same person with concurrent fluid and imaging measurements, so their results can often be used interchangeably. However, discordance may occur between the three biomarker measures.

Plasma tau phosphorylated at threonine 181 (p-tau181) has recently emerged as an accessible, scalable, and highly specific biomarker for AD [5–8], which showed strong associations with CSF and PET tau indicators [9, 10]. The disagreement between this novel biomarker and CSF p-tau181 or tau-PET has been proposed recently [11]. However, it is still unclear whether this discordance could affect disease severity and whether plasma p-tau181 could be used to detect early pathology in AD. As for CSF p-tau and tau-PET, they may capture different aspects of tau pathology [12, 13]. CSF p-tau exhibits higher sensitivity and thus better reflects “disease state,” while tau-PET shows continuous accumulation and thus indicates “disease stage” [14, 15]. Besides, CSF p-tau181 may become abnormal earlier than tau-PET, which was demonstrated previously among Alzheimer’s Disease Neuroimaging Initiative (ADNI) participants [16]. Herein, we investigated the discrepancies between plasma p-tau181 and CSF p-tau181 or tau-PET. We hypothesized that discordant plasma and CSF or PET tau indicators denoted different stages of disease severity.

## Methods

### Study design

Data used in the preparation for this article were derived from the ADNI database (http://adni.loni.usc.edu) [17, 18]. The ADNI was launched in 2003 as a public-private partnership with the primary goal of testing the effectiveness of integrating neuroimaging, clinical, biological, and neuropsychological markers in measuring the progression of mild cognitive impairment (MCI) and early AD. All ADNI individuals were recruited from over 50 sites across the USA and Canada.

### Participants

We extracted all demographic information from the latest merged document “ADNIMERGE.csv” updated on May 24, 2019. To detect the early accrual of tau proteins, we only included non-demented subjects diagnosed as cognitively normal (CN) controls or MCI. For detailed diagnostic criteria, see www.adni-info.org. The tau biomarker data from 724 participants was available. These participants were followed up periodically, with visits every 3 months for the first year, followed by half-year visits. Only 668 subjects who had both plasma and CSF p-tau181 data at baseline were included in the plasma/CSF group. There were 304 ADNI subjects receiving at least 1 plasma p-tau181 measurement and 1 tau-PET scan. Among them, 245 subjects had both assessments within a 36-month interval and were included in the plasma/PET group. Only 44 subjects had concurrent plasma and PET tau information.

### Plasma assessments

Plasma p-tau181 was measured using an assay developed in-house on a Simoa HD-X (Quanterix, Billerica, MA, USA) instrument in the Clinical Neurochemistry Laboratory, University of Gothenburg, Sweden [7]. The assay utilizes a combination of two monoclonal antibodies (Tau12 and AT270) and measures N-terminal to mid-domain forms of p-tau181. All plasma samples were measured in a single batch [7]. The within-run variations and between-run variations ranged consistently below 15%. We extracted data from the latest available dataset (“UGOTPTAU181_06_18_20.csv”). The cutoff value for plasma p-tau181 was determined among ADNI participants based on the Youden index, using receiver operating characteristic (ROC) analysis (Additional file 1: Appendix 1). This analysis identified a threshold of 18.849 pg/ml that best distinguished amyloid-β (Aβ)-negative CN individuals (158 persons) from Aβ-positive AD dementia patients (119 persons). The area under the curve (AUC) was 0.844 (95% confidence interval (CI) = 0.795–0.892) with 76% sensitivity and 85% specificity. More than 94% of Aβ-negative CN subjects were included in the analyses of our paper. Since this study only focused on non-demented subjects, all Aβ-positive AD patients were excluded from our analyses. Aβ status was determined by CSF Aβ42 levels or Aβ-PET SUVRs if the participant lacked CSF Aβ42 data. In addition, we also tried to calculate the threshold in an independent ADNI set. The ROC analysis identified a threshold of 13.556 pg/ml that distinguished 9 Aβ-negative CN persons from 119 Aβ-positive AD patients. The AUC was 0.686 (95% CI = 0.411–0.962) with 90% sensitivity and 67% specificity. Considering the limited sample in the independent set and the bad diagnostic performance, we did not utilize this cutoff value. A future larger independent ADNI set is needed to calculate the threshold of plasma p-tau181.

### CSF measurements

CSF samples were collected and shipped on dry ice to the ADNI Biomarker core laboratory. Aliquots (0.5 mL) were prepared from these samples and stored in polypropylene tubes at −80 °C. For plasma/CSF categories, we used CSF data from ADNI 1 and 2 and GO. All CSF concentrations were measured using automated Roche Elecsys and cobas e 601 immunoassay analyzer systems [19]. All CSF biomarker assays were performed in duplicate and averaged. The coefficients of variation obtained on the CSF analytes in each batch and between batches were < 15%. The cutoffs of CSF Aβ42 and CSF p-tau181 have been previously set at 1098 pg/ml [16, 20] and 26.64 pg/ml [16], respectively.

### PET image processing

A detailed description of Aβ (florbetapir, or [^18^F] AV45) and tau (flortaucipir, or [^18^F] AV1451) PET image acquisition and processing can be found at http://adni.loni.usc.edu/datasamples/pet/. The mean standard uptake value ratio (SUVR) was calculated relative to a reference region. For Aβ-PET, the region of interest (ROI) was a composite region comprising the whole cerebellum, brainstem/pons, and subcortical white matter. This composite region had more reliable longitudinal florbetapir results in ADNI compared to utilizing only the cerebellum as a reference region [21]. The SUVR cutoff of Aβ-PET was 0.79 [21, 22]. As for tau-PET, the composite region was made up of bilateral entorhinal, amygdala, fusiform, inferior temporal, and middle temporal cortices. The meta-ROI SUVR threshold of tau-PET without partial volume correction was 1.37 [16]. The flortaucipir-PET SUVR values in Braak stage ROIs were also extracted.

### Neuroimaging and cognition

For structural magnetic resonance image (MRI) brain scans, automated volume measures were obtained with the FreeSurfer software (http://surfer.nmr.mgh.harvard.edu/fswiki) [23]. The ROI we selected was the hippocampus. Estimated intracranial volume (ICV) was used to adjust ROI for head size variation based on covariance.

Cognitive evaluations were performed using composite scores reflecting memory [24] and executive function (EF) [25].

### Grouping of subjects

As for CSF tau status, we used p-tau181 that is generally believed to reveal tau pathology (total-tau being considered a more general indicator of neurodegeneration) [26]. Since plasma total-tau performs relatively poorly in AD settings [5, 27], we chose plasma p-tau181 as the tau biomarker. Regarding tau-PET, we chose the composite region, which was described to be AD specific [28]. Based on the described cutoffs, we categorized the participants as positive or negative on each modality. This resulted in 2 groups: plasma/CSF group (plasma−/CSF−, plasma+/CSF−, plasma−/CSF+, and plasma+/CSF+) and plasma/PET group (plasma−/PET−, plasma+/PET−, plasma−/PET+, and plasma+/PET+). According to concordance status, the participants were also classified into concordant-negative, discordant, and concordant-positive groups. The Aβ status was determined based on CSF Aβ42 levels. Since some participants lacked the CSF Aβ42 measurement, their Aβ status was accessed by Aβ-PET. The results barely changed when only CSF Aβ42 was used to determine Aβ status.

### Statistical analyses

Group differences were assessed using chi-square tests for categorical data or Kruskal-Wallis tests for continuous variables, followed by post hoc analyses where appropriate. Linear associations were analyzed by Spearman correlations. Cohen’s kappa statistic was used to quantify agreements between dichotomous (+/−) tau measurements. We investigated tau biomarker groups’ relation to CSF Aβ42 and Aβ-PET, as well as the flortaucipir uptake in Braak stage ROIs, using general linear models where log10 transformation was performed to approximate a normal distribution. For longitudinal analyses, time point 0 corresponding to the plasma collection visit was regarded as the reference time. Cognitive decline and brain atrophy over time were compared in linear mixed-effects (LME) models with random slopes and intercepts. The time-by-group interaction in LME models predicted changes in the specified outcomes (memory scores, EF scores, and hippocampal volumes). To access the risk of clinical disease progression (cognitive decline), we constructed unadjusted Kaplan-Meier plots. Progressive cognitive deterioration was defined as follows: (1) CN subjects converted to MCI or AD, or their global Clinical Dementia Rating (CDR) scores rose to ≥ 0.5 and (2) MCI subjects met any one of the following three criteria: during the follow-up visit, the MMSE score was lower than the MMSE score of time point 0 by > 3 points; MCI subjects converted to AD dementia at follow-up; or they got a MMSE score < 24 during the follow-up [29–31]. Multivariate Cox proportional-hazard models estimated the association between biomarker group and the risk of cognitive deterioration. The outcome of the model was time to cognitive decline. Hazard ratios (HRs) were reported, and the assumption of proportional hazards was tested through Schoenfield residuals. Furthermore, we compared cognitive deterioration across different tau biomarker groups in individuals with and without signs of Aβ pathology separately. Covariates of the aforementioned models comprised age, sex, years of education, and *APOE* ε4. Specifically, we adjusted for age, gender, *APOE* ε4, and ICV when analyzing hippocampal volumes. In the plasma/PET group, we additionally adjusted for the time span between plasma and PET assessments. The main results have not changed when the time delay was not corrected. Sensitivity analyses were also conducted.

Statistical significance was defined as *P* < 0.05 (two-sided). Statistical analyses were completed using the R software (version 3.5.1).

## Results

### Disagreements of AD tau biomarkers

**Fig. 1:**
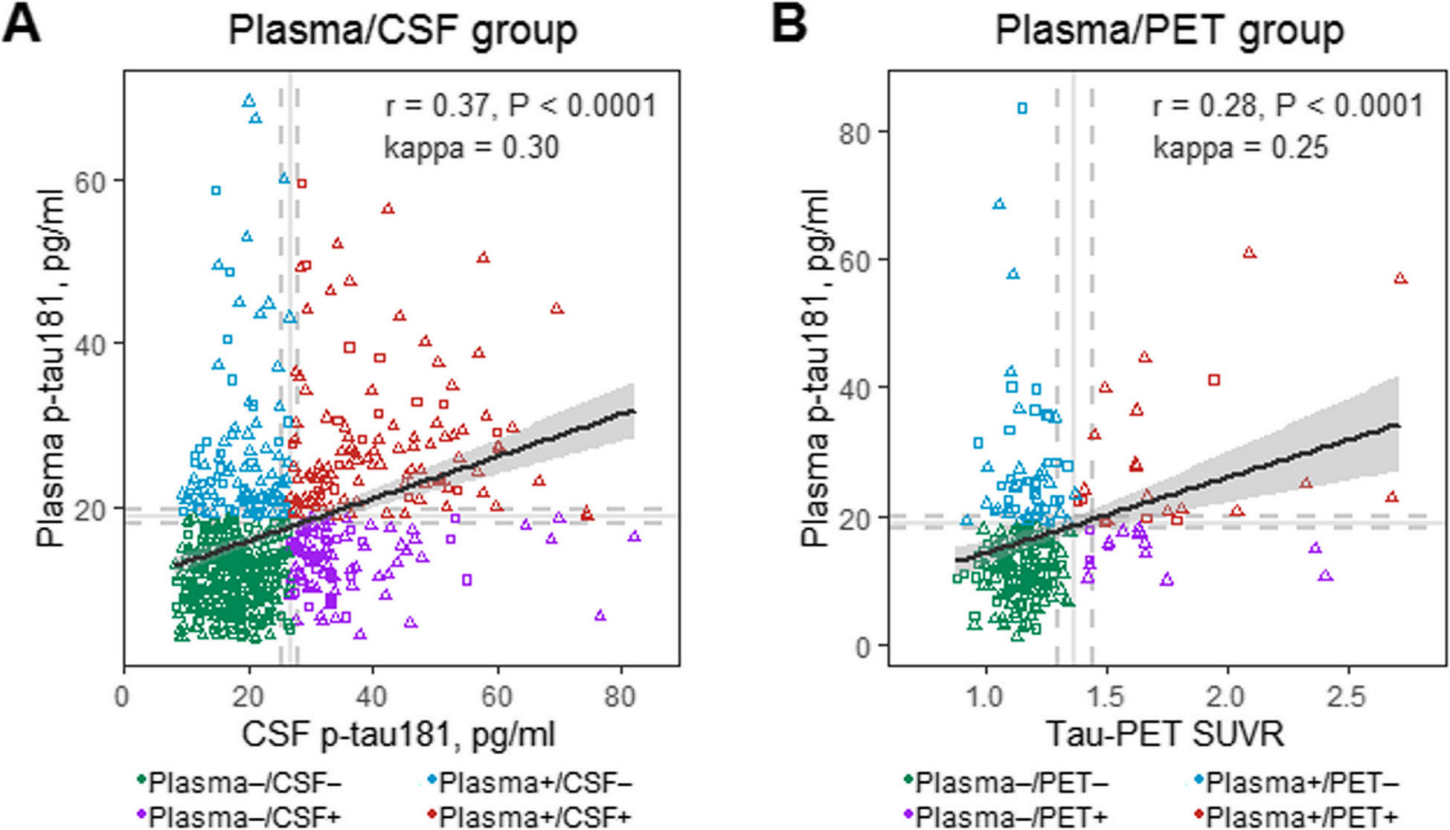
Scatterplots reflecting concordance status between tau biomarkers. **a** Plasma/CSF group: plasma p-tau181 versus CSF p-tau181; **b** Plasma/PET group: plasma p-tau181 versus AV1451 PET. Color code represents tau biomarker categories, and shape indicates the clinical diagnosis. Solid lines delineate the thresholds for tau biomarkers. Dashed lines delineate a ± 5% interval from the thresholds. Abbreviations: CN, cognitively normal; CSF, cerebrospinal fluid; MCI, mild cognitive impairment; PET, positron emission tomography; p-tau181, tau phosphorylated at threonine 181; SUVR, standard uptake value ratio

First, we assessed the concordance status among plasma, CSF, and PET tau measures. The subject classification discordance across the plasma/CSF group and plasma/PET group reached 31% and 29%, respectively. Isolated plasma or CSF p-tau181 positivity was seen in several cases, but isolated AV1451 PET positivity was very rare. In detail, there were 668 participants in the plasma/CSF tau group, of whom 345 participants were classified as plasma−/CSF− (51.6%), 104 as plasma+/CSF− (15.6%), 102 as plasma−/CSF+ (15.3%), and 117 as plasma+/CSF+ (17.5%). The plasma/PET tau group comprised 245 participants [151 plasma−/PET− (61.6%), 55 plasma+/PET− (22.4%), 15 plasma−/PET+ (6.1%), and 24 plasma+/PET+ (9.8%)] (Table 1). Despite the small size, we included the interesting plasma−/PET+ group in our analyses. Removal of persons within 5% of tau biomarker thresholds minimized the size of the plasma−/PET+ group to only 10 persons (Additional file 1: Appendix 2), further highlighting the rarity of isolated PET-positive individuals. The correlations between continuous tau biomarkers were modest (Spearman coefficient *r* < 0.40), so were the agreements between dichotomous (+/−) tau measures (kappa < 0.40) (Fig. 1).

**Table 1:** Sample characteristics

Characteristics	Plasma/CSF group					Plasma/PET group				
	Plasma−/CSF−	Plasma+/CSF−	Plasma−/CSF+	Plasma+/CSF+	*P*	Plasma−/PET–	Plasma+/PET−	Plasma−/PET+	Plasma+/PET+	*P*
Numbers (%)	345 (51.6)	104 (15.6)	102 (15.3)	117 (17.5)		151 (61.6)	55 (22.4)	15 (6.1)	24 (9.8)	
Age (years)	70.34 (6.58)	73.06 (7.18)	73.90 (7.13)	74.28 (6.72)	**<0.001**	70.43 (6.42)	73.56 (7.12)	73.05 (5.64)	73.03 (7.45)	**0.011**
Female (%)	170 (49.3)	39 (37.5)	52 (51.0)	61 (52.1)	0.113	74 (49.0)	21 (38.2)	7 (46.7)	15 (62.5)	0.239
Educational years	16.43 (2.51)	16.34 (2.78)	16.50 (2.56)	16.15 (2.59)	0.735	16.32 (2.73)	16.49 (2.68)	16.53 (2.23)	15.38 (2.75)	0.360
*APOE* Ɛ4 (%)	95 (27.5)	45 (43.3)	52 (51.0)	81 (69.2)	**<0.001**	42 (27.8)	24 (43.6)	10 (66.7)	11 (45.8)	**0.005**
Diagnosis										
CN	157 (45.5)	34 (32.7)	38 (37.3)	23 (19.7)	**<0.001**	83 (55.0)	29 (52.7)	3 (20.0)	7 (29.2)	**0.011**
MCI	188 (54.5)	70 (67.3)	64 (62.7)	94 (80.3)		68 (45.0)	26 (47.3)	12 (80.0)	17 (70.8)	
CSF Aβ42 (pg/ml)	1347.37 (568.07)	1080.68 (591.37)	1210.44 (732.29)	831.29 (409.36)	**<0.001**	1470.49 (600.76)	1121.27 (607.10)	892.15 (487.98)	776.61 (383.27)	**<0.001**
Aβ-PET SUVR	0.78 (0.08)	0.86 (0.15)	0.90 (0.14)	1.01 (0.11)	**<0.001**	0.77 (0.07)	0.81 (0.09)	0.99 (0.12)	0.98 (0.13)	**<0.001**
Aβ+ (%)	129 (37.4)	66 (63.5)	65 (63.7)	99 (84.6)	**<0.001**	44 (31.2)	27 (57.4)	11 (78.6)	22 (95.7)	**<0.001**
Plasma to PET (years)	–	–	–	–	–	1.60 (0.98)	1.62 (0.97)	1.60 (1.12)	1.83 (1.13)	0.759

Continuous variables were presented as means (standard deviations (SDs)), and categorical variables were presented as numbers (percent)

*Abbreviations*: *Aβ*, amyloid-β; *CN*, cognitively normal; *CSF*, cerebrospinal fluid; *MCI*, mild cognitive impairment; *PET*, positron emission tomography; *SUVR*, standard uptake value ratio

In addition, 20 participants had all 3 modalities. Among them, the overall concordance and discordance were both 50% [7 plasma−/CSF−/PET− (35%), 3 plasma+/CSF−/PET− (15%), 3 plasma−/CSF+/PET− (15%), 0 plasma−/CSF−/PET+ (0%), 1 plasma+/CSF+/PET− (5%), 0 plasma+/CSF−/PET+ (0%), 3 plasma−/CSF+/PET+ (15%), and 3 plasma+/CSF+/PET+ (15%)]. Due to the limited sample size, we did not study the cross-sectional and longitudinal characteristics of each subgroup. Future larger cohorts are needed to do this.

### Cross-sectional characteristics

**Fig. 2:**
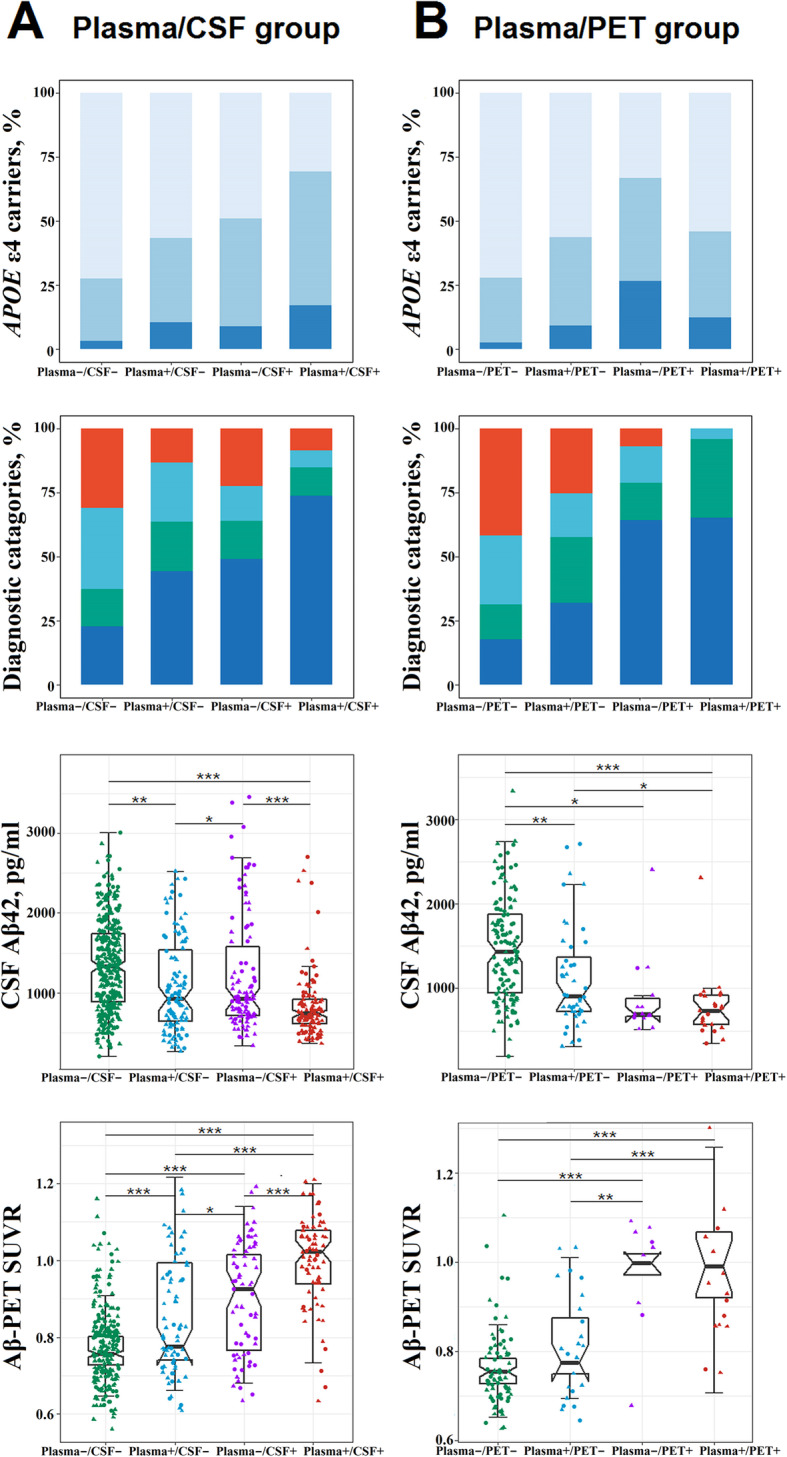
Cross-sectional characteristics of tau biomarker groups. **a** Plasma/CSF group. **b** Plasma/PET group. The frequency of *APOE* ε4 allele, Aβ pathology, and MCI, as well as the Aβ burden (reflected by CSF Aβ42 concentrations and Aβ-PET SUVRs), was compared. Significance levels for group comparisons: *P < 0.05, **P < 0.01, ***P < 0.001. Abbreviations: Aβ, amyloid-β; CN, cognitively normal; CSF, cerebrospinal fluid; MCI, mild cognitive impairment; PET, positron emission tomography; SUVR, standard uptake value ratio

Next, baseline characteristics were compared between the groups. All plasma/CSF participants had similar sex ratios and education levels but differed by age, so did plasma/PET persons (Table 1 and Additional file 1: Appendix 3). The follow-up time ranged from 6 to 156 months. The time delay between PET and plasma assessments did not show significant differences between the groups. As for plasma p-tau181 versus CSF p-tau181, the prevalence of *APOE* ε4 allele, Aβ pathology, or MCI was highest for subjects who were positive for both markers, lowest for subjects negative for both, and intermediate for the 2 discordant groups (Fig. 2a). This trend remained when it comes to Aβ burden as measured by CSF Aβ42 and Aβ-PET. The prevalence of *APOE* ε4 allele, Aβ pathology, or MCI did not differ between the plasma+/CSF− and plasma−/CSF+ groups, while the plasma+/CSF− group showed lower CSF Aβ42 (*P* = 0.04) and lower Aβ-PET (*P* = 0.03) levels than the plasma−/CSF+ group. Concerning plasma/PET categories (Fig. 2b and Additional file 1: Appendix 4), as expected, individuals who were PET-positive (plasma+/PET+ and plasma−/PET+) had greater proportions of *APOE* ε4 carriers and persons with Aβ pathology or MCI, lower CSF Aβ42 or higher Aβ-PET values, and elevated flortaucipir retention in Braak ROIs relative to those concordant-negative. Of note, compared with those plasma+/PET−, those PET-positive demonstrated a greater prevalence of Aβ pathology or MCI, more Aβ-PET tracer uptake, and increased tracer uptake across Braak stage I through VI ROIs.

### Differences in longitudinal clinical outcomes

**Fig. 3:**
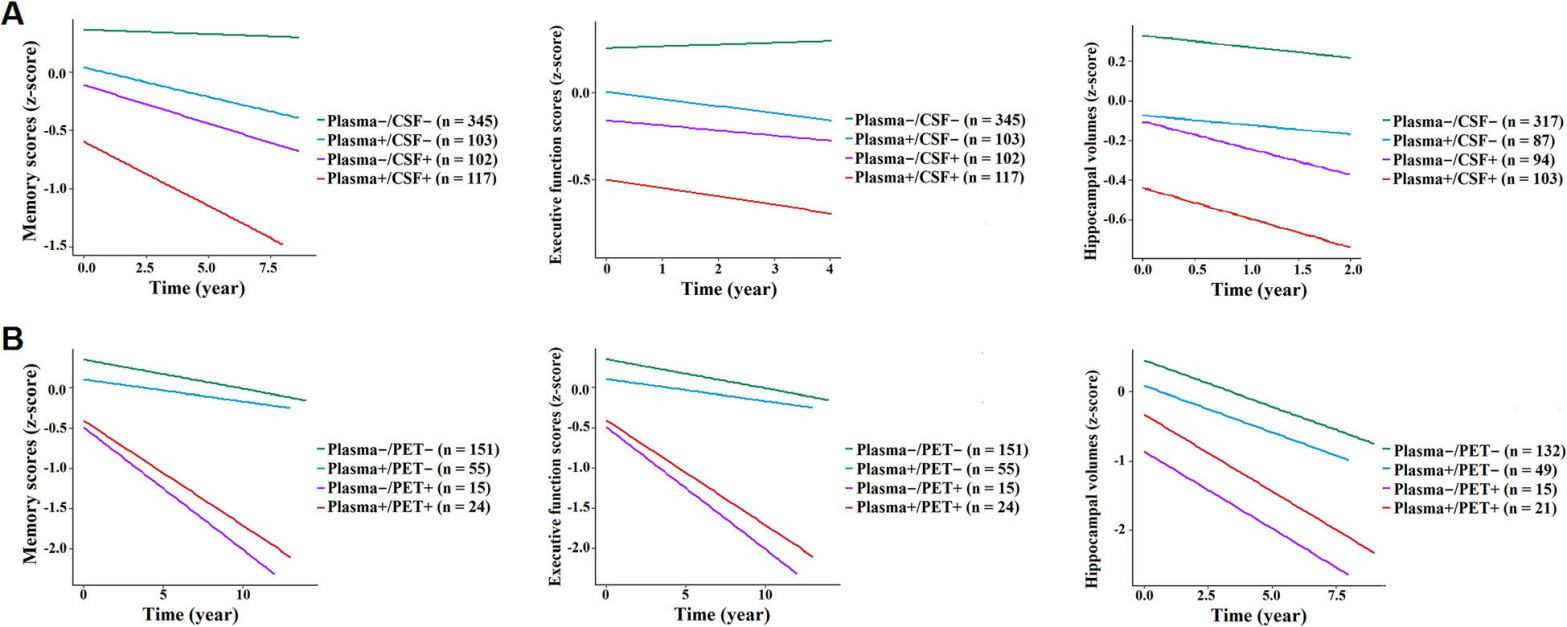
Longitudinal changes in cognitive scores and hippocampal volumes. **a** Plasma/CSF group. **b** Plasma/PET group. All outcome variables were standardized to *z*-scores to facilitate comparisons between modalities. Alterations in clinical outcomes over time were modeled using linear mixed-effects regression with data from different visits. Abbreviations: CSF, cerebrospinal fluid; PET, positron emission tomography

Longitudinally, we used cognitive scales and MRI scans to investigate the declines in brain function (cognitive function reductions) and structure (brain volume loss). Available data at each follow-up visit were listed in Additional file 1: Appendix 5. Differences between every pair of groups were demonstrated by estimates with standard error (SE) and *P* values (Additional file 1: Appendix 6). As for plasma p-tau181 versus CSF p-tau181 (Fig. 3a), the concordant-positive group showed faster clinical progression than the remaining 3 groups (plasma−/CSF−, plasma+/CSF−, and plasma−/CSF+), except for the differences in the hippocampal atrophy rates between plasma+/CSF+ and plasma−/CSF+. Both discordant groups (plasma+/CSF− and plasma−/CSF+) exhibited greater clinical progression than the concordant− group. However, no remarkable differences were detected between the two discordant groups in terms of memory scores, EF scores, and hippocampal volumes.

In plasma/PET categories (Fig. 3b), as compared with concordant-negative individuals, those PET-positive (plasma+/PET+ and plasma−/PET+) showed greater change rates of clinical outcomes, whereas this was not true for those plasma+/PET−. It is worth noting that concordant-positive individuals had accelerated cognitive decline and hippocampal atrophy than those plasma+/PET−, while they performed identically to those plasma−/PET+. Besides, plasma−/PET+ individuals had a more rapid decline in memory function than those plasma+/PET−.

### Prediction of disease progression for each biomarker profile

**Fig. 4:**
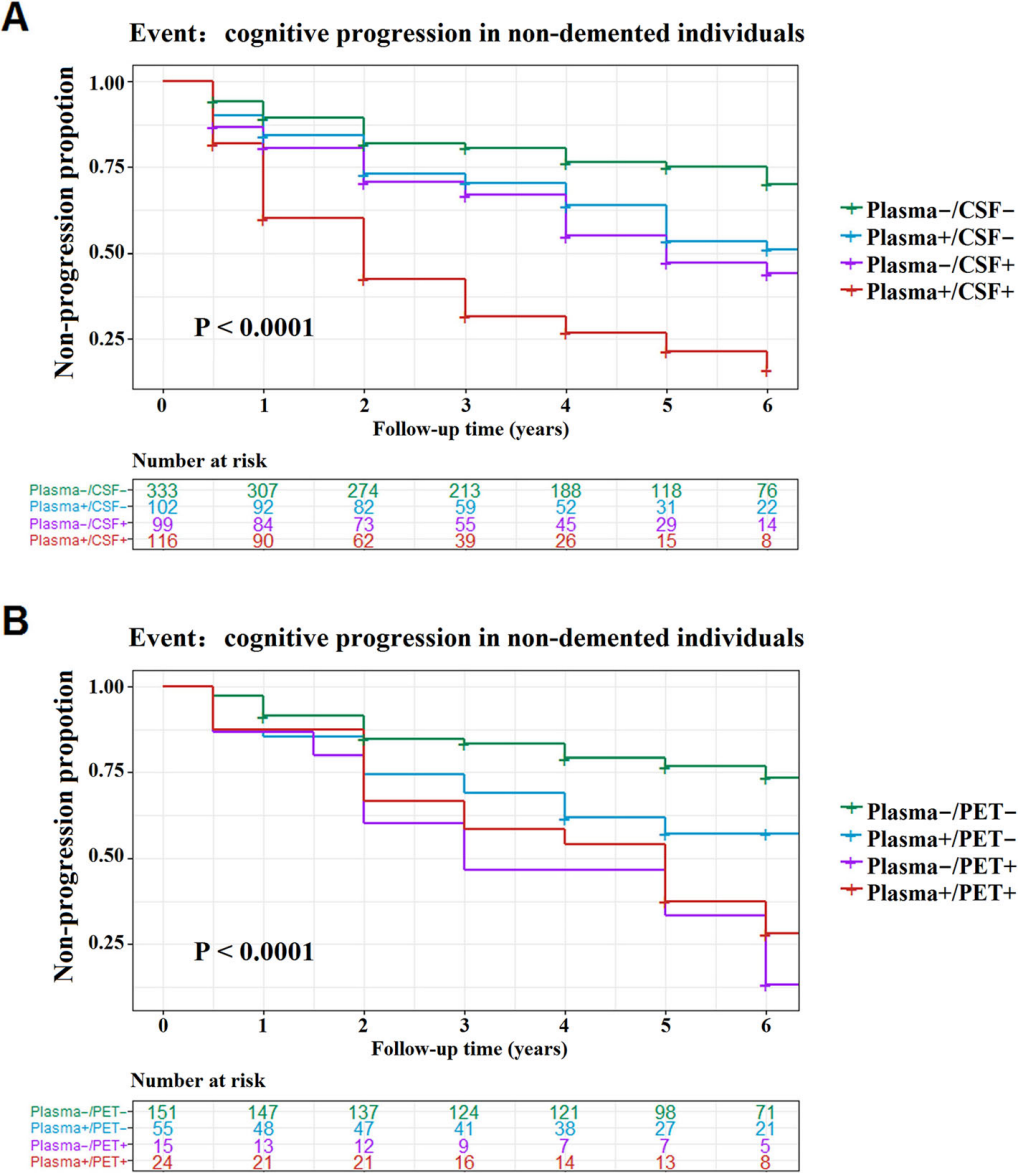
Kaplan-Meier curves showing the cumulative probability of clinical disease progression. **a** Plasma/CSF group. **b** Plasma/PET group. The numbers of individuals at risk at different follow-up time points were presented. Survival time was calculated according to the intervals from the baseline evaluation to the time points of clinical progression. Abbreviations: CSF, cerebrospinal fluid; PET, positron emission tomography

To explore whether tau biomarker categories indicated distinct states of AD pathological progression, we inquired whether these groups had divergent trajectories of cognitive conversion. As delineated in Kaplan-Meier curves regarding plasma/CSF categories (Fig. 4a), all tau+ groups on either measure tended to progress faster than the concordant-negative group. This finding was robust in Cox regression for the discordant groups (plasma+/CSF−: HR = 1.54, 95% CI = 1.05–2.27; plasma−/CSF+: HR = 1.89, 95% CI = 1.30–2.77) as well as the concordant-positive group (HR = 3.72, 95% CI = 2.62–5.26). Those concordant-positive also had an increased risk of cognitive conversion, relative to those plasma+/CSF− (HR = 2.42, 95% CI = 1.63–3.61) and plasma−/CSF+ (HR = 2.03, 95% CI = 1.39–2.97). However, we did not observe any difference in conversion risk between the two discordant groups.

As for plasma p-tau181 versus AV1451 PET (Fig. 4b), as expected, both concordant-positive (HR = 2.76, 95% CI = 1.54–4.95) and discordant (plasma+/PET−: HR = 1.77, 95% CI = 1.07–2.95; plasma−/PET+: HR = 4.25, 95% CI = 2.17–8.32) groups showed a higher conversion risk in comparison with the concordant-negative group. Noteworthy, plasma−/PET+ individuals were more likely to progress than plasma+/PET− persons, with intergroup differences approaching statistical significance (HR = 1.99, 95% CI = 0.98–4.03, *P* = 0.057). However, the progression risk did not differ between the discordant and concordant-positive groups.

The trajectories of cognitive conversion among the CN and MCI population alone were similar to those among the general population (Additional file 1: Appendix 7), demonstrating that the analyses were not affected by clinical diagnosis. Considering the small sample sizes in certain groups, it is necessary to validate our results in larger samples.

### Subgroup analyses stratified by Aβ status

To further investigate whether tau biomarker groups’ relation to cognitive decline (measured by longitudinal changes in cognitive scores, and risk of cognitive conversion) is affected by Aβ status, we repeated the above analyses in individuals with and without signs of Aβ pathology separately. From Additional file 1: Appendix 8, we could see that the main results derived from the combined sample barely changed in individuals with Aβ pathology, whereas no significant intergroup differences were observed in persons without Aβ pathology.

### Sensitivity analyses

Sensitivity analyses were further performed to test the robustness of our primary results. First, we repeated the main analyses after removing persons within 5% of the plasma p-tau181, CSF p-tau181, and tau-PET thresholds. The results were essentially unchanged (Additional file 1: Appendix 9), suggesting that results were not driven by the borderline cases. Second, we considered data only from participants who had plasma p-tau181 and tau-PET assessments at the same visit, albeit the sample sizes in some plasma/PET groups were too small to evaluate the findings (Additional file 1: Appendix 10). When considering data from participants who underwent plasma p-tau181 and tau-PET assessments within a 12-month interval, we reached similar conclusions to the main analyses (Additional file 1: Appendix 11). Third, we used an alternative ROI (entorhinal cortex, one of the earliest regions of AD-related tau pathology [32]) for tau PET in the plasma/PET group, the main results barely changed (Additional file 1: Appendix 12). Fourth, using a previous cutoff (plasma p-tau181 concentrations > 17.7 pg/ml were considered positive) generated by the Youden index obtained from the ADNI [11], we attained similar results (Additional file 1: Appendix 13). This finding verified the reliability of our analyses.

## Discussion

By characterizing individuals with AD tau biomarker discrepancies, this study indicates that (1) individuals with isolated abnormal plasma p-tau181 and normal CSF p-tau181 showed similarly reduced cognitive capacities to persons with normal plasma p-tau181 and abnormal CSF p-tau181; (2) those PET-positive appeared to have a poorer cognitive function, as well as increased tau-PET binding in Braak stage ROIs, irrespective of the status of plasma p-tau181; and (3) changes in plasma and CSF p-tau181 were associated with established Aβ pathology. Taken together, plasma and CSF p-tau181 abnormalities in relation to amyloidosis may occur simultaneously in the course of the disease, prior to AV1451 PET positivity.

Understanding the discrepancies between tau measurements is essential. We found discordance between the tau measures was approximately 30%. Overall, our data provide support for this hypothesis: plasma p-tau181 elevates as early as CSF p-tau181 in the course of the disease. The plasma+/CSF− and plasma−/CSF+ groups accounted for identical proportions. These two discordant profiles were intermediate between plasma−/CSF− and plasma+/CSF+ in terms of disease severity, as manifested by the frequency of Aβ pathology or MCI, rates of cognitive decline, and risk of cognitive conversion. Importantly, plasma+/CSF− and plasma−/CSF+ participants had comparable cognitive performance, which is in agreement with previous findings using continuous measures [8, 10]. The elevations of p-tau181 in the plasma and CSF may thus reflect similar underlying pathological processes characterized by early tau abnormality. Another piece of evidence is that plasma p-tau181 becomes abnormal before significant tau deposition is detected by AV1451 PET. Plasma+/PET− participants were substantially more common than plasma−/PET+ persons. This suggests that plasma abnormality alone may indicate the more typical intermediate state in AD pathogenesis. Within plasma/PET categories, as expected, PET-positive individuals exhibited a greater frequency of Aβ pathology or MCI, Aβ burden, and cognitive deterioration than those concordant-negative. Of note, discordant subjects with normal PET had slower cognitive decline, relative to PET-positive persons. It can be concluded that those PET-positive appear to have poorer cognitive capacities, regardless of the plasma status. Besides, PET-positive individuals may be closer to AD dementia than those discordant with isolated plasma+. Furthermore, PET-positive individuals had increased tau-PET binding in Braak stage ROIs, when compared with both plasma+/PET− and plasma−/PET− participants. This finding suggested that plasma tau measures, rather than AV1451 PET, reflected earlier tau pathological changes. To sum up, plasma p-tau181 abnormality may occur as early as CSF p-tau181 abnormality, followed by AV1451 PET positivity. Accordingly, the exploration of different approaches to the characterization of tau pathology at the single-subject level is encouraged in the future.

**Fig. 5:**
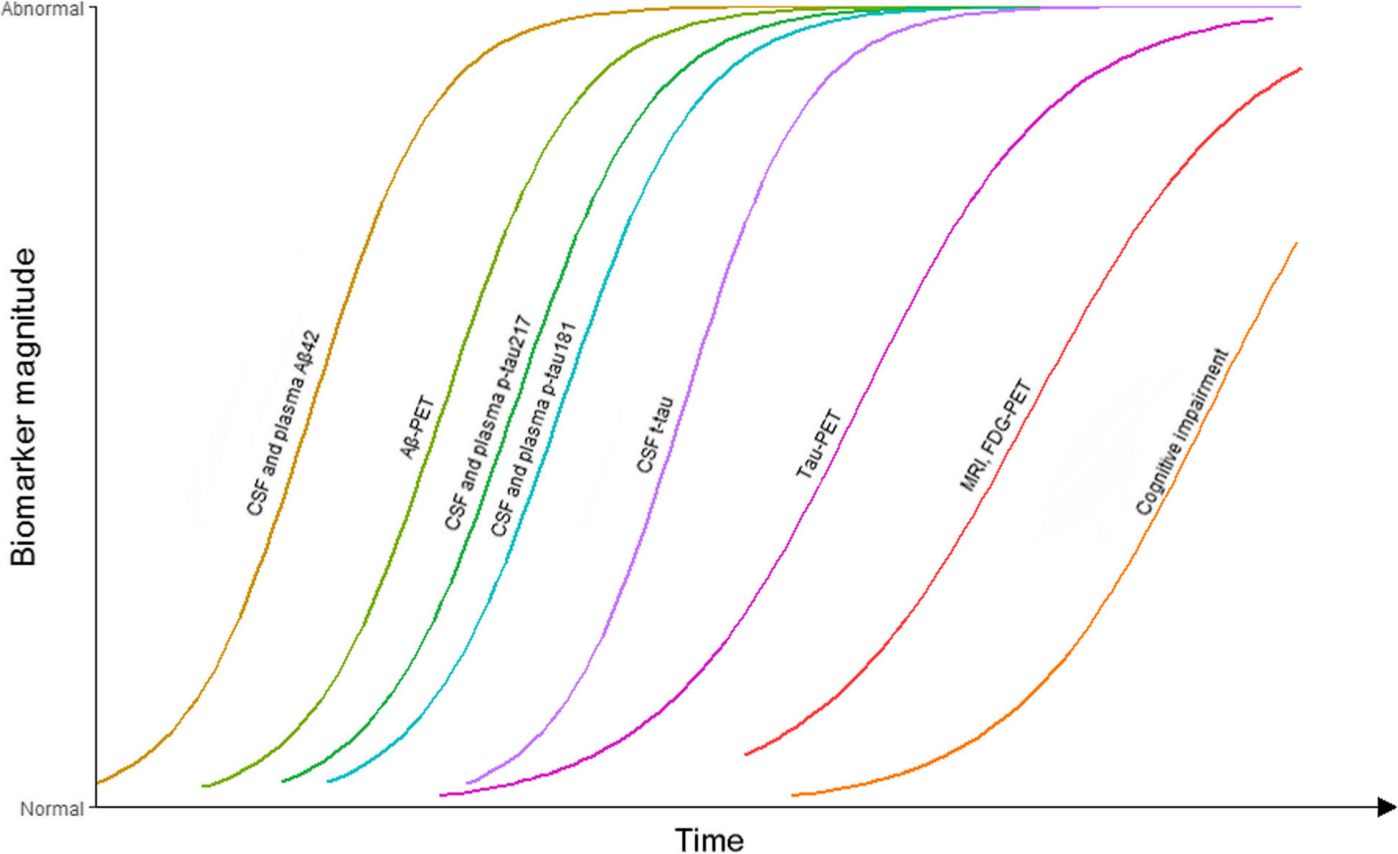
A model of the temporal pattern of AD-related biomarker abnormalities. Combining our and previous findings [33, 36], we delineate an approximative sequence of how different biomarkers change during the time course. Aβ biomarkers become abnormal first, which is shortly followed by alterations of soluble tau. Shortly thereafter, tau-PET turns positive. Taking into account personal reserves and vulnerability factors, we acknowledge that large interindividual differences in the timing of different events may exist. Abbreviations: Aβ, amyloid-β; AD, Alzheimer’s disease; CSF, cerebrospinal fluid; FDG, fluorodeoxyglucose; MRI, magnetic resonance imaging; PET, positron emission tomography; p-tau181, tau phosphorylated at threonine 181; p-tau217, tau phosphorylated at threonine 217; t-tau, total-tau

The present study extends prior evidence by showing that the significant changes of tau biomarkers are tightly linked to established Aβ pathology. The finding that changes of plasma or CSF p-tau181 occur early was seen only in individuals with signs of Aβ deposition. This suggests that Aβ pathology induces alterations in the metabolism of soluble tau, which seems necessary for the formation of tau deposits [33]. Consistent with the recently proposed model of AD [5], our derived temporal pattern of biomarker abnormalities extends the amyloid cascade hypothesis [34]. Specifically, as a neuronal reaction to Aβ aggregation [35], the elevation in soluble tau (plasma and CSF p-tau181) appears upstream, while tau-PET as the most direct indicator of tau tangle pathology turns abnormal later [5]. Consequently, there may be a window of opportunity for interference and treatment against tau pathology in Aβ-positive individuals before flortaucipir-PET abnormality. Furthermore, our work encourages the refinement of biomarker-based classification of AD (“ATN” scheme) [26], as some individuals may be classified as “Aβ positive, plasma or CSF p-tau181 positive, and tau-PET negative.” Synthesizing the findings in this study, together with previous literatures [33, 36], a model of biomarker trajectories in AD may therefore be updated (Fig. 5).

Our findings have clear implications for the use of plasma or CSF tau biomarkers in early AD. First, the observation that prominent changes in soluble tau occur as a function of Aβ deposition indicates the high specificity of plasma or CSF p-tau181 to AD neuropathology, as previous studies have corroborated [7, 33, 37]. Second, plasma p-tau181 may be a reliable alternative to CSF p-tau181 in detecting those likely to be tau positive. Besides, simultaneous assessments of plasma and CSF p-tau181 may provide complementary information to clinicians in certain prognostic (i.e., predicting clinical progression in individuals without dementia) scenarios. Third, plasma and CSF tau biomarkers are not completely interchangeable. For instance, those plasma−/CSF+ are more likely to show signs of Aβ-PET while those plasma+/CSF− are more likely to show signs of CSF Aβ42, suggesting that plasma and CSF p-tau181 may differ in their association with Aβ pathology. And this needs to be further explored in the future. Fourth, plasma p-tau181 and CSF p-tau181 may reach abnormality early before tau-PET turns positive, supporting their role as early biomarkers of AD pathophysiology. Anti-Aβ treatments have so far failed to effectively curb disease progression [5], spurring on the development and testing of tau-target therapies [38]. Accordingly, identifying individuals with altered soluble tau biomarkers but without widespread tau deposition on PET may be of vital importance [33], for example, for epidemiological or interventional studies, to investigate the effects of risk factors, protective factors, and disease-modifying interventions, and for clinical trials, to monitor treatment efficacy (i.e., blocking the disease cascade to minimize the development of pathology and symptoms). Furthermore, both CSF and PET measurements have notable hurdles. They are invasive, time-consuming, and expensive, and they may have side effects and poor availability, particularly in primary care [8, 39]. Consequently, the less invasive, time-saving, cost-effective, easily accessible, and highly specific plasma p-tau181 may become a preferable tool in future clinical practice and trials [5–7], especially when the access to CSF or PET testing is limited [40].

## Limitations

The primary strength of this study is the large prospective cohort design with long follow-up, based on which the cognitive trajectories in non-demented individuals were well characterized. An additional strength is that the main results were robust after sensitivity analyses. Nonetheless, some caveats should be emphasized. First, the number of participants with tau-PET scans was relatively small, and the results need to be replicated in larger cohorts. The findings of the present study should also be verified for other tau-PET tracers. Second, the robust threshold of plasma p-tau181 requires validation in other cohorts with different populations. And our results require validation using other cutoffs. Besides, a larger independent set of samples is needed to calculate the threshold of plasma p-tau181. Third, although the batch analyses performed in this study may have lower variability than the sequential analyses of samples, the performance of biomarkers may be affected by analytical variability in real-life settings.

## Conclusions

To conclude, plasma and CSF p-tau181 abnormalities associated with amyloidosis occur simultaneously in the progression of AD pathogenesis and related cognitive decline, before tau-PET turns positive. Plasma p-tau181 could be a desirable alternative and complement to CSF p-tau181 in detecting early tau deposition, and its abnormality alone may indicate a suitable stage for starting disease-modifying treatments or interventions for modifiable risk factors. It is foreseeable that in the field of AD, blood tests will be attractive in future clinical practice and trials.

## Supplementary Information

**Additional file 1: Appendix 1.** Flowchart and ROC analysis for plasma p-tau181. **Appendix 2.** Sample characteristics after removing borderline cases. **Appendix 3.** Intergroup comparisons of baseline characteristics. **Appendix 4.** Comparison of flortaucipir binding across tau biomarker groups. **Appendix 5**: Available longitudinal data for linear mixed-effects models. **Appendix 6.** Longitudinal analyses of clinical outcomes. **Appendix 7.** Results of subgroup analyses stratified by clinical diagnosis. **Appendix 8.** Results of subgroup analyses stratified by Aβ status. **Appendix 9.** Results after removing borderline cases. **Appendix 10.** Results using concurrent tau measures. **Appendix 11.** Results using plasma p-tau181 and tau-PET assessments within a 12-month interval. **Appendix 12.** Results using an alternative ROI (entorhinal cortex). **Appendix 13.** Results using previous cut-off for plasma p-tau181.

## Data Availability

The data used and analyzed in this study are available from the corresponding authors on reasonable request.

## Abbreviations

Aβ: Amyloid-β
AD: Alzheimer’s disease
ADNI: Alzheimer’s Disease Neuroimaging Initiative
AUC: Area under the curve
CDR: Clinical Dementia Rating
CI: Confidence interval
CN: Cognitively normal
CSF: Cerebrospinal fluid
EF: Executive function
HR: Hazard ratio
ICV: Intracranial volume
LME: Linear mixed-effects
MCI: Mild cognitive impairment
MMSE: Mini-Mental State Examination
MRI: Magnetic resonance image
PET: Positron emission tomography
p-tau181: Tau phosphorylated at threonine 181
ROC: Receiver operating characteristics
ROI: Region of interests
SE: Standard error
SUVR: Standard uptake value ratio
