# Supplementary material for: Characterization of Alzheimer’s tau biomarker discordance using plasma, CSF, and PET
Source: Alzheimers Res Ther. 2021 May 4;13:93. doi: 10.1186/s13195-021-00834-3 (PMC8094494; doi:10.1186/s13195-021-00834-3)
Supplement: Supplementary file 1 — Additional file 1: Appendix 1. Flowchart and ROC analysis for plasma p-tau181. Appendix 2. Sample characteristics after removing borderline cases. Appendix 3. Intergroup comparisons of baseline characteristics. Appendix 4. Comparison of flortaucipir binding across tau biomarker groups. Appendix 5: Available longitudinal data for linear mixed-effects models. Appendix 6. Longitudinal analyses of clinical outcomes. Appendix 7. Results of subgroup analyses stratified by clinical diagnosis. Appendix 8. Results of subgroup analyses stratified by Aβ status. Appendix 9. Results after removing borderline cases. Appendix 10. Results using concurrent tau measures. Appendix 11. Results using plasma p-tau181 and tau-PET assessments within a 12-month interval. Appendix 12. Results using an alternative ROI (entorhinal cortex). Appendix 13. Results using previous cut-off for plasma p-tau181. [file 13195_2021_834_MOESM1_ESM.docx]

**Content:**

**Appendix 1: Flowchart and ROC analysis for plasma p-tau181……………………...……...….…..1**

**Appendix 2: Sample characteristics after removing borderline cases………………….………..….3**

**Appendix 3: Intergroup comparisons of baseline characteristics……....................………….…......4**

**Appendix 4: Comparison of flortaucipir binding across tau biomarker groups………….………..5**

**Appendix 5: Available longitudinal data for linear mixed-effects models………………….…...…..6**

**Appendix 6: Longitudinal analyses of clinical outcomes…………………….………………..……..8**

**Appendix 7: Results of subgroup analyses stratified by clinical diagnosis……………………...….9**

**Appendix 8: Results of subgroup analyses stratified by Aβ status……………………...............…13**

**Appendix 9: Results after removing borderline cases……………………………………………....21**

**Appendix 10: Results using concurrent tau measures………..…………………………………......27**

**Appendix 11: Results using plasma p-tau181 and tau-PET assessments within a 12-month interval……..………………………………………………………………………………………......30**

**Appendix 12: Results using an alternative ROI (entorhinal cortex) ……..………………………..33**

**Appendix 13: Results using previous cut-off for plasma p-tau181……………………………........36**

Appendix 1: Flowchart and ROC analysis for plasma p-tau181

Flowchart:


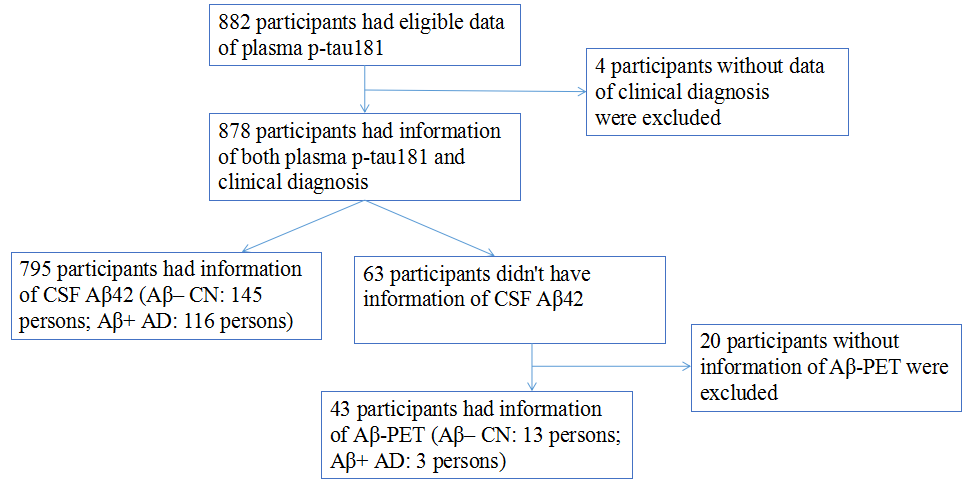


ROC analysis:


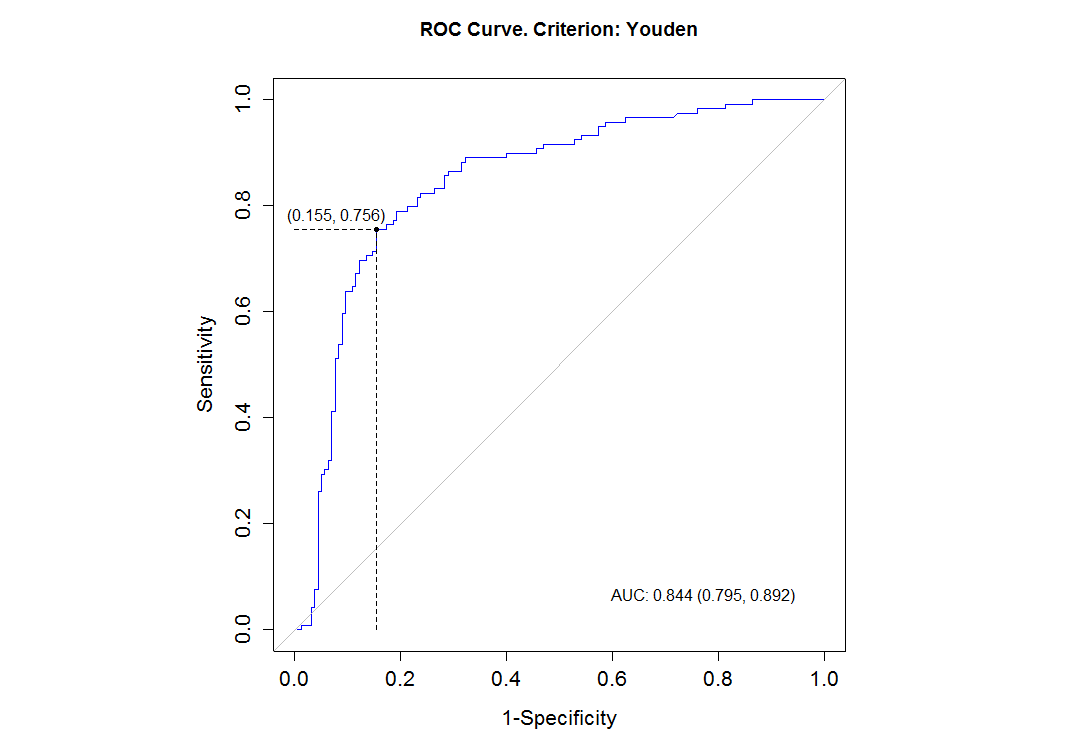


|  | Estimate | LCI | UCI |
| --- | --- | --- | --- |
| Cutoff value | 18.849 | - | - |
| Sensitivity (%) | 0.7563025 | 0.6691131 | 0.8303183 |
| Specificity (%) | 0.8451613 | 0.7784283 | 0.8982017 |
| PPV (%) | 0.7894737 | 0.707059 | 0.8553418 |
| NPV (%) | 0.81875 | 0.7464115 | 0.8795482 |

Note: The cutoff for plasma p-tau181 was determined based on the Youden index, using ROC analyses. The analyses identified a threshold of 18.849 pg/ml that best distinguished Aβ– CN individuals (158 persons) from Aβ+ AD patients (119 persons). To obtain the cutoff for plasma p-tau181, we extracted the CSF data measured using the multiplex xMAP Luminex platform (Luminex Corp, Austin, TX) with the INNOBIA AlzBio3 kit (Innogenetics) and the automated Roche Elecsys and cobas e 601 immunoassay analyzer systems. When using the former measurement, Aβ+ was defined as CSF Aβ42 levels ≤ 192 pg/ml (Reference: Jagust WJ, Landau SM, Shaw LM, Trojanowski JQ, Koeppe RA, Reiman EM, et al. Relationships between biomarkers in aging and dementia. Neurology. 2009;73:1193-9). When using the latter measurement, Aβ+ was defined as CSF Aβ42 levels ≤ 1098 pg/ml (Reference: Meyer PF, Pichet Binette A, Gonneaud J, Breitner JCS, Villeneuve S. Characterization of Alzheimer Disease Biomarker Discrepancies Using Cerebrospinal Fluid Phosphorylated Tau and AV1451 Positron Emission Tomography. JAMA Neurol. 2020;77:508-16). The greatest ROC area under the curve (AUC) for plasma p-tau181 was obtained when we used the former CSF measurement. That is to say, by defining the Aβ status as CSF Aβ42 levels ≤ 192 pg/ml, we got a plasma p-tau181 threshold of 18.849 pg/ml, with better diagnostic performance.

Abbreviations: Aβ = amyloid-β, AD = Alzheimer’s disease, AUC = area under the curve, CN = cognitively normal, CSF = cerebrospinal fluid, LCI = lower confidence interval, NPV = negative predictive value, PET = positron emission tomography, PPV = positive predictive value, p-tau181 = tau phosphorylated at threonine 181, ROC = receiver operating characteristic, UCI = upper confidence interval.

Appendix 2: Sample characteristics after removing borderline cases

| Characteristics | Plasma/CSF group | | | | |  | Plasma/PET group | | | | |
| --- | --- | --- | --- | --- | --- | --- | --- | --- | --- | --- | --- |
|  | Plasma– | Plasma+ | Plasma– | Plasma+ | P |  | Plasma– | Plasma+ | Plasma– | Plasma+ | P |
|  | /CSF– | /CSF– | /CSF+ | /CSF+ |  |  | /PET– | /PET– | /PET+ | /PET+ |  |
| Numbers | 317 | 78 | 84 | 100 |  |  | 139 | 44 | 10 | 16 |  |
| Age (years) | 70.18 (6.43) | 73.34 (7.07) | 74.64 (7.15) | 74.18 (6.49) | **<0.001** |  | 70.54 (6.34) | 73.92 (7.62) | 74.02 (5.51) | 70.96 (7.81) | **0.020** |
| Female (%) | 156 (49.2) | 24 (30.8) | 44 (52.4) | 53 (53.0) | **0.011** |  | 67 (48.2) | 16 (36.4) | 3 (30.0) | 11 (68.8) | 0.100 |
| Educational years | 16.42 (2.52) | 16.62 (2.81) | 16.45 (2.59) | 16.06 (2.61) | 0.513 |  | 16.41 (2.69) | 16.55 (2.57) | 15.80 (2.04) | 14.81 (2.51) | 0.110 |
| *APOE* Ɛ4 (%) | 85 (26.8) | 35 (44.9) | 44 (52.4) | 74 (74.0) | **<0.001** |  | 35 (25.2) | 19 (43.2) | 8 (80.0) | 9 (56.2) | **<0.001** |
| Diagnosis |  |  |  |  |  |  |  |  |  |  |  |
| CN (%) | 139 (43.8) | 24 (30.8) | 32 (38.1) | 22 (22.0) | **0.001** |  | 80 (57.6) | 25 (56.8) | 1 (10.0) | 1 (6.2) | **<0.001** |
| MCI (%) | 178 (56.2) | 54 (69.2) | 52 (61.9) | 78 (78.0) |  |  | 59 (42.4) | 19 (43.2) | 9 (90.0) | 15 (93.8) |  |

Continuous variables were presented as means (standard deviations (SDs)), and categorical variables were presented as numbers (percents).

Abbreviations: CN = cognitively normal, CSF = cerebrospinal fluid, MCI = mild cognitive impairment, PET = positron emission tomography.

Appendix 3: Intergroup comparisons of baseline characteristics

|  | P-value | |
| --- | --- | --- |
|  | Plasma/CSF group | Plasma/PET group |
| Age (years) | a,b,c | g |
| Female (%) | - | - |
| Educational years | - | - |
| *APOE* Ɛ4 (%) | a,b,c,e,f | g,h |
| Diagnosis | a,c,e,f | h,i |
| CSF Aβ42 | a,c,d,f | g,h,i,k |
| Aβ-PET SUVR | a,b,c,d,e,f | h,i,j,k |

Note: ^a^ Plasma–/CSF– vs Plasma+/CSF–; P < 0.05.

^b^ Plasma–/CSF– vs Plasma–/CSF+; P < 0.05.

^c^ Plasma–/CSF– vs Plasma+/CSF+; P < 0.05.

^d^ Plasma+/CSF– vs Plasma–/CSF+; P < 0.05.

^e^ Plasma+/CSF– vs Plasma+/CSF+; P < 0.05.

^f^ Plasma–/CSF+ vs Plasma+/CSF+; P < 0.05.

^g^ Plasma–/PET– vs Plasma+/PET–; P < 0.05.

^h^ Plasma–/PET– vs Plasma–/PET+; P < 0.05.

^i^ Plasma–/PET– vs Plasma+/PET+; P < 0.05.

^j^ Plasma+/PET– vs Plasma–/PET+; P < 0.05.

^k^ Plasma+/PET– vs Plasma+/PET+; P < 0.05.

^l^ Plasma–/PET+ vs Plasma+/PET+; P < 0.05.

Abbreviations: Aβ = amyloid-β, CSF = cerebrospinal fluid, PET = positron emission tomography, SUVR = standard uptake value ratio.

Appendix 4: Comparison of flortaucipir binding across tau biomarker groups


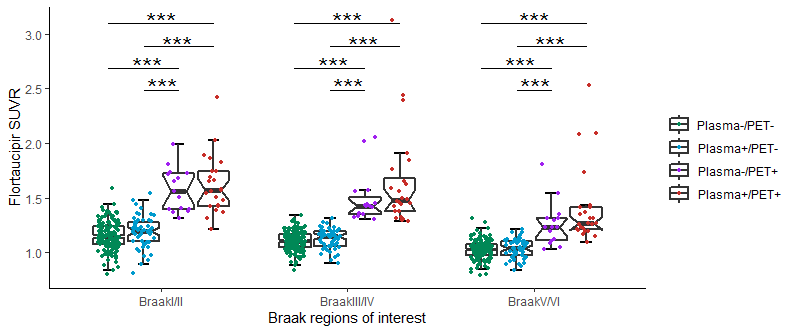


Abbreviations: PET = positron emission tomography, SUVR = standard uptake value ratio.

**Note:** * represents p-value < 0.05, ** represents p-value < 0.01, and *** represents p-value < 0.001.

Appendix 5: Available longitudinal data for linear mixed-effects models

Memory scores

|  | 0m | 12m | 24m | 36m | 48m | 60m | 72m | 84m | 96m | 108m | 120m | 132m | 144m | 156m | 168m |
| --- | --- | --- | --- | --- | --- | --- | --- | --- | --- | --- | --- | --- | --- | --- | --- |
| Plasma–/CSF– | 345 | 277 | 290 | 161 | 200 | 93 | 123 | 69 | 46 | 5 | 0 | 0 | 0 | 0 | 0 |
| Plasma+/CSF– | 103 | 94 | 87 | 53 | 58 | 24 | 39 | 21 | 11 | 1 | 0 | 0 | 0 | 0 | 0 |
| Plasma–/CSF+ | 102 | 86 | 84 | 49 | 52 | 30 | 25 | 19 | 11 | 4 | 0 | 0 | 0 | 0 | 0 |
| Plasma+/CSF+ | 117 | 104 | 91 | 62 | 58 | 30 | 12 | 10 | 3 | 0 | 0 | 0 | 0 | 0 | 0 |
|  |  |  |  |  |  |  |  |  |  |  |  |  |  |  |  |
| Plasma–/PET– | 151 | 129 | 141 | 90 | 134 | 77 | 124 | 74 | 60 | 17 | 14 | 14 | 11 | 10 | 2 |
| Plasma+/PET– | 55 | 49 | 55 | 36 | 48 | 33 | 33 | 28 | 15 | 6 | 7 | 7 | 5 | 4 | 0 |
| Plasma–/PET+ | 15 | 14 | 15 | 12 | 14 | 9 | 12 | 8 | 5 | 2 | 1 | 2 | 1 | 0 | 0 |
| Plasma+/PET+ | 24 | 22 | 23 | 17 | 21 | 14 | 15 | 12 | 5 | 2 | 3 | 1 | 2 | 1 | 0 |

Executive function scores

|  | 0m | 12m | 24m | 36m | 48m | 60m | 72m | 84m | 96m | 108m | 120m | 132m | 144m | 156m | 168m | 180m |
| --- | --- | --- | --- | --- | --- | --- | --- | --- | --- | --- | --- | --- | --- | --- | --- | --- |
| Plasma–/CSF– | 345 | 277 | 288 | 157 | 198 | 90 | 120 | 66 | 45 | 5 | 0 | 0 | 0 | 0 | 0 | 0 |
| Plasma+/CSF– | 103 | 94 | 87 | 53 | 55 | 23 | 37 | 19 | 11 | 1 | 0 | 0 | 0 | 0 | 0 | 0 |
| Plasma–/CSF+ | 102 | 86 | 84 | 49 | 52 | 30 | 25 | 19 | 10 | 4 | 0 | 0 | 0 | 0 | 0 | 0 |
| Plasma+/CSF+ | 117 | 104 | 89 | 60 | 56 | 27 | 11 | 10 | 3 | 0 | 0 | 0 | 0 | 0 | 0 | 0 |
|  |  |  |  |  |  |  |  |  |  |  |  |  |  |  |  |  |
| Plasma–/PET– | 151 | 129 | 140 | 88 | 135 | 77 | 122 | 73 | 59 | 17 | 14 | 14 | 11 | 10 | 2 | 1 |
| Plasma+/PET– | 55 | 49 | 55 | 35 | 47 | 31 | 33 | 27 | 15 | 6 | 7 | 7 | 5 | 4 | 0 | 0 |
| Plasma–/PET+ | 15 | 14 | 15 | 12 | 14 | 9 | 12 | 6 | 5 | 2 | 0 | 1 | 1 | 0 | 0 | 0 |
| Plasma+/PET+ | 24 | 22 | 23 | 17 | 21 | 14 | 15 | 12 | 5 | 2 | 3 | 1 | 2 | 1 | 0 | 0 |

Hippocampal volumes

|  | 0m | 12m | 24m | 36m | 48m | 60m | 72m | 84m | 96m | 108m | 120m |
| --- | --- | --- | --- | --- | --- | --- | --- | --- | --- | --- | --- |
| Plasma–/CSF– | 317 | 245 | 233 | 39 | 59 | 1 | 0 | 0 | 0 | 0 | 0 |
| Plasma+/CSF– | 87 | 74 | 69 | 15 | 17 | 1 | 0 | 0 | 0 | 0 | 0 |
| Plasma–/CSF+ | 94 | 72 | 75 | 13 | 16 | 1 | 0 | 0 | 0 | 0 | 0 |
| Plasma+/CSF+ | 103 | 95 | 84 | 15 | 14 | 0 | 0 | 0 | 0 | 0 | 0 |
|  |  |  |  |  |  |  |  |  |  |  |  |
| Plasma–/PET– | 132 | 114 | 120 | 28 | 41 | 13 | 16 | 7 | 6 | 3 | 0 |
| Plasma+/PET– | 49 | 41 | 48 | 10 | 15 | 2 | 6 | 3 | 3 | 0 | 0 |
| Plasma–/PET+ | 15 | 13 | 13 | 4 | 5 | 1 | 2 | 0 | 1 | 0 | 0 |
| Plasma+/PET+ | 21 | 17 | 21 | 4 | 6 | 2 | 1 | 2 | 1 | 1 | 0 |

Note: m represents follow-up months.

Abbreviations: CSF = cerebrospinal fluid, PET = positron emission tomography.

Appendix 6: Longitudinal analyses of clinical outcomes

Plasma/CSF group:

|  | Memory scores | | |  | Executive function scores | | |  | Hippocampal volumes | | |
| --- | --- | --- | --- | --- | --- | --- | --- | --- | --- | --- | --- |
|  | β | SE | P |  | β | SE | P |  | β | SE | P |
| Plasma–/CSF– | Reference | Reference | Reference |  | Reference | Reference | Reference |  | Reference | Reference | Reference |
| Plasma+/CSF– | -0.054 | 0.016 | **0.001** |  | -0.032 | 0.017 | 0.058 |  | -0.036 | 0.016 | **0.030** |
| Plasma–/CSF+ | -0.081 | 0.017 | **<0.001** |  | -0.075 | 0.017 | **<0.001** |  | -0.077 | 0.016 | **<0.001** |
| Plasma+/CSF+ | -0.175 | 0.017 | **<0.001** |  | -0.172 | 0.018 | **<0.001** |  | -0.094 | 0.016 | **<0.001** |
|  |  |  |  |  |  |  |  |  |  |  |  |
| Plasma+/CSF– | Reference | Reference | Reference |  | Reference | Reference | Reference |  | Reference | Reference | Reference |
| Plasma–/CSF+ | -0.025 | 0.024 | 0.298 |  | -0.044 | 0.026 | 0.088 |  | -0.043 | 0.025 | 0.083 |
| Plasma+/CSF+ | -0.124 | 0.024 | **<0.001** |  | -0.146 | 0.026 | **<0.001** |  | -0.063 | 0.024 | **0.008** |
|  |  |  |  |  |  |  |  |  |  |  |  |
| Plasma–/CSF+ | Reference | Reference | Reference |  | Reference | Reference | Reference |  | Reference | Reference | Reference |
| Plasma+/CSF+ | -0.099 | 0.027 | **<0.001** |  | -0.103 | 0.028 | **<0.001** |  | -0.019 | 0.022 | 0.395 |

Plasma/PET group:

|  | Memory scores | | |  | Executive function scores | | |  | Hippocampal volumes | | |
| --- | --- | --- | --- | --- | --- | --- | --- | --- | --- | --- | --- |
|  | β | SE | P |  | β | SE | P |  | β | SE | P |
| Plasma–/PET– | Reference | Reference | Reference |  | Reference | Reference | Reference |  | Reference | Reference | Reference |
| Plasma+/PET– | -0.020 | 0.016 | 0.201 |  | -0.030 | 0.016 | 0.062 |  | -0.001 | 0.020 | 0.954 |
| Plasma–/PET+ | -0.124 | 0.026 | **<0.001** |  | -0.094 | 0.027 | **0.001** |  | -0.070 | 0.033 | **0.034** |
| Plasma+/PET+ | -0.140 | 0.022 | **<0.001** |  | -0.167 | 0.022 | **<0.001** |  | -0.119 | 0.027 | **<0.001** |
|  |  |  |  |  |  |  |  |  |  |  |  |
| Plasma+/PET– | Reference | Reference | Reference |  | Reference | Reference | Reference |  | Reference | Reference | Reference |
| Plasma–/PET+ | -0.100 | 0.031 | **0.002** |  | -0.063 | 0.035 | 0.074 |  | -0.066 | 0.041 | 0.109 |
| Plasma+/PET+ | -0.112 | 0.027 | **<0.001** |  | -0.140 | 0.030 | **<0.001** |  | -0.121 | 0.035 | **0.001** |
|  |  |  |  |  |  |  |  |  |  |  |  |
| Plasma–/PET+ | Reference | Reference | Reference |  | Reference | Reference | Reference |  | Reference | Reference | Reference |
| Plasma+/PET+ | -0.009 | 0.045 | 0.850 |  | -0.076 | 0.051 | 0.149 |  | -0.053 | 0.044 | 0.233 |

Abbreviations: CSF = cerebrospinal fluid, PET = positron emission tomography, SE = standard error.

Appendix 7: Results of subgroup analyses stratified by clinical diagnosis


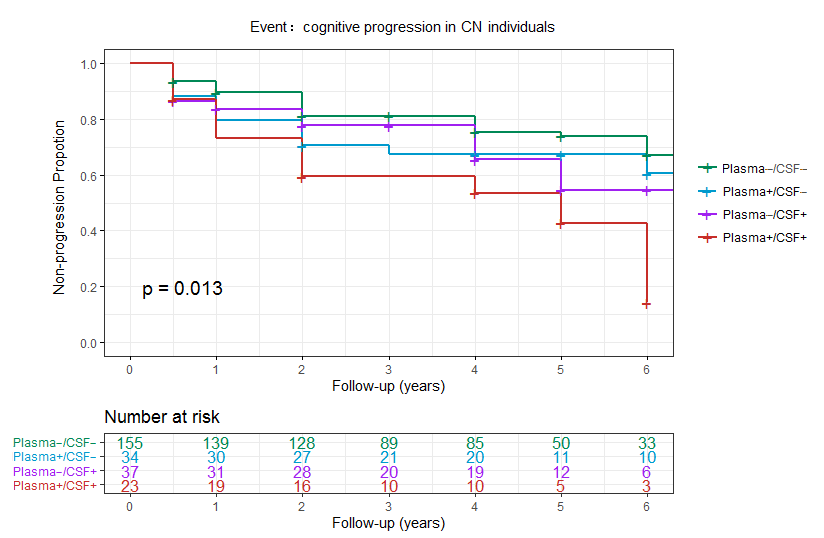


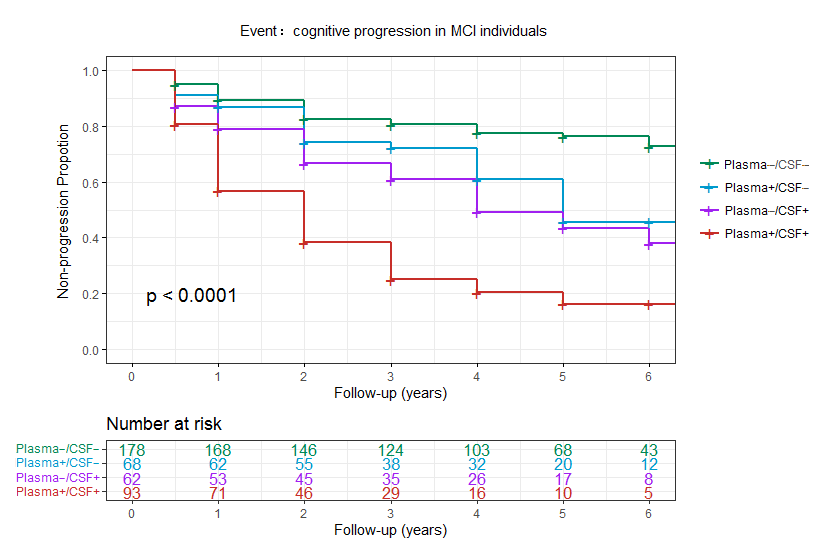


|  | CN individuals | | | |  | MCI individuals | | | |
| --- | --- | --- | --- | --- | --- | --- | --- | --- | --- |
|  | HR | LCI | UCI | P |  | HR | LCI | UCI | P |
| Plasma–/CSF– | Reference | Reference | Reference | Reference |  | Reference | Reference | Reference | Reference |
| Plasma+/CSF– | 1.183 | 0.603 | 2.323 | 0.625 |  | 1.769 | 1.084 | 2.888 | **0.023** |
| Plasma–/CSF+ | 1.283 | 0.673 | 2.449 | 0.449 |  | 2.235 | 1.376 | 3.631 | **0.001** |
| Plasma+/CSF+ | 2.195 | 1.109 | 4.345 | **0.024** |  | 4.507 | 2.901 | 7.001 | **<0.001** |
|  |  |  |  |  |  |  |  |  |  |
| Plasma+/CSF– | Reference | Reference | Reference | Reference |  | Reference | Reference | Reference | Reference |
| Plasma–/CSF+ | 0.988 | 0.438 | 2.228 | 0.976 |  | 1.212 | 0.723 | 2.029 | 0.466 |
| Plasma+/CSF+ | 1.894 | 0.843 | 4.257 | 0.122 |  | 2.504 | 1.571 | 3.992 | **<0.001** |
|  |  |  |  |  |  |  |  |  |  |
| Plasma–/CSF+ | Reference | Reference | Reference | Reference |  | Reference | Reference | Reference | Reference |
| Plasma+/CSF+ | 1.843 | 0.809 | 4.196 | 0.146 |  | 2.102 | 1.352 | 3.268 | **0.001** |


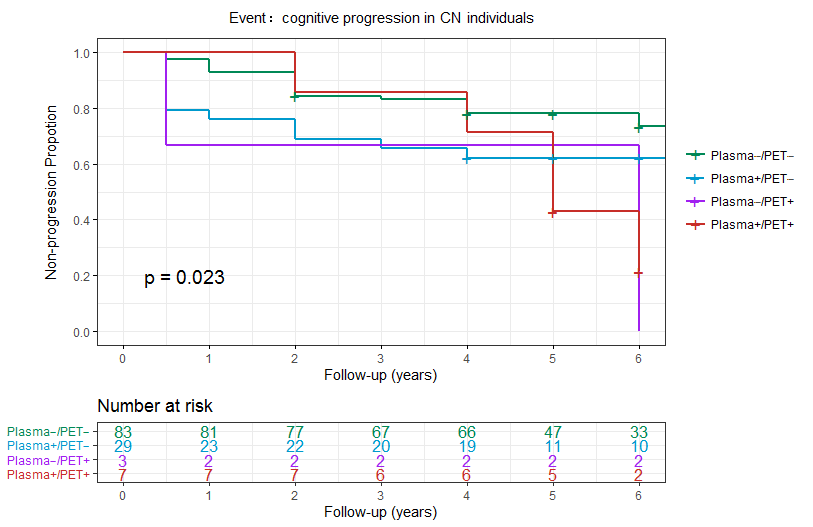


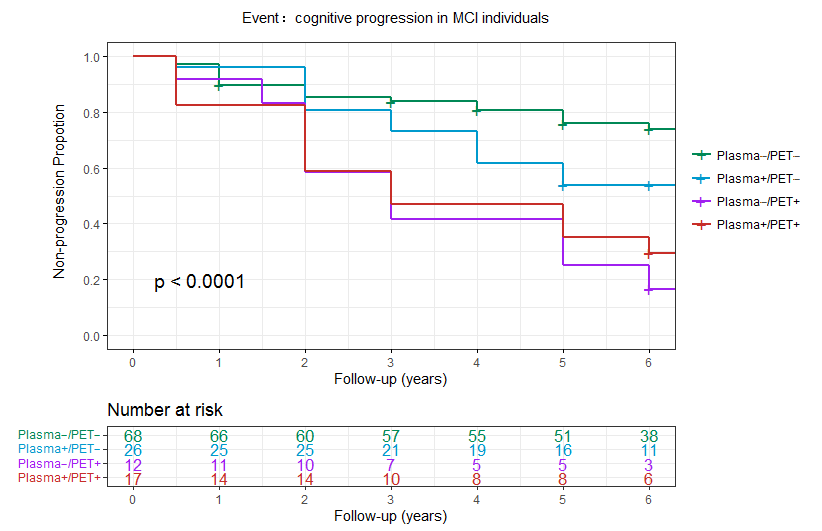


|  | CN individuals | | | |  | MCI individuals | | | |
| --- | --- | --- | --- | --- | --- | --- | --- | --- | --- |
|  | HR | LCI | UCI | P |  | HR | LCI | UCI | P |
| Plasma–/PET– | Reference | Reference | Reference | Reference |  | Reference | Reference | Reference | Reference |
| Plasma+/PET– | 1.538 | 0.739 | 3.203 | 0.250 |  | 1.939 | 0.937 | 4.015 | 0.074 |
| Plasma–/PET+ | 5.086 | 1.302 | 19.867 | **0.019** |  | 4.208 | 1.798 | 9.847 | **0.001** |
| Plasma+/PET+ | 3.087 | 1.117 | 8.530 | **0.030** |  | 2.892 | 1.320 | 6.333 | **0.008** |
|  |  |  |  |  |  |  |  |  |  |
| Plasma+/PET– | Reference | Reference | Reference | Reference |  | Reference | Reference | Reference | Reference |
| Plasma–/PET+ | 1.214 | 0.252 | 5.849 | 0.809 |  | 2.164 | 0.910 | 5.146 | 0.081 |
| Plasma+/PET+ | 1.379 | 0.427 | 4.458 | 0.591 |  | 1.884 | 0.779 | 4.557 | 0.160 |
|  |  |  |  |  |  |  |  |  |  |
| Plasma–/PET+ | Reference | Reference | Reference | Reference |  | Reference | Reference | Reference | Reference |
| Plasma+/PET+ | 1.914 | 0.089 | 41.018 | 0.678 |  | 0.759 | 0.275 | 2.092 | 0.594 |

Abbreviations: CN = cognitively normal, CSF = cerebrospinal fluid, HR = hazard ratio, LCI = lower confidence interval, MCI = mild cognitive impairment, PET = positron emission tomography, UCI = upper confidence interval.

Appendix 8: Results of subgroup analyses stratified by Aβ status

Plasma/CSF group:

Longitudinal analyses:

A+ individuals:


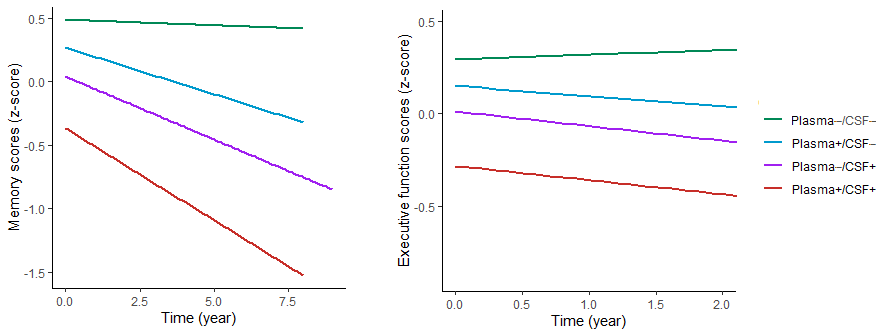


A– individuals:


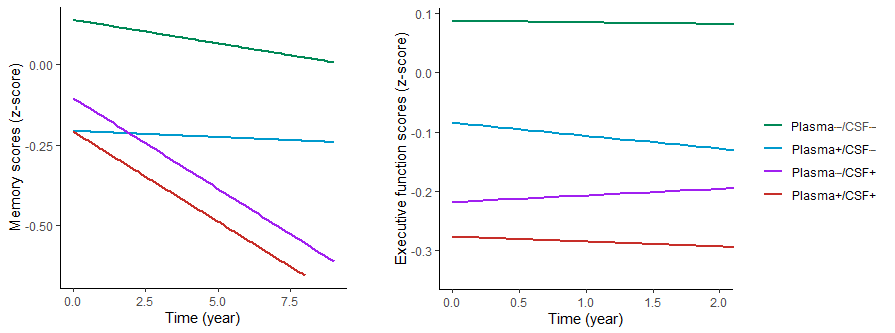


|  | Memory scores in A+ individuals | | |  | Memory scores in A– individuals | | |
| --- | --- | --- | --- | --- | --- | --- | --- |
|  | β | SE | P |  | β | SE | P |
| Plasma–/CSF– | Reference | Reference | Reference |  | Reference | Reference | Reference |
| Plasma+/CSF– | -0.065 | 0.026 | **0.012** |  | -0.018 | 0.022 | 0.421 |
| Plasma–/CSF+ | -0.115 | 0.027 | **<0.001** |  | -0.022 | 0.022 | 0.327 |
| Plasma+/CSF+ | -0.191 | 0.024 | **<0.001** |  | -0.043 | 0.032 | 0.175 |
|  |  |  |  |  |  |  |  |
| Plasma+/CSF– | Reference | Reference | Reference |  | Reference | Reference | Reference |
| Plasma–/CSF+ | -0.049 | 0.033 | 0.134 |  | 0.001 | 0.034 | 0.974 |
| Plasma+/CSF+ | -0.129 | 0.030 | **<0.001** |  | -0.026 | 0.042 | 0.545 |
|  |  |  |  |  |  |  |  |
| Plasma–/CSF+ | Reference | Reference | Reference |  | Reference | Reference | Reference |
| Plasma+/CSF+ | -0.082 | 0.034 | **0.018** |  | -0.029 | 0.045 | 0.525 |

|  | Executive function scores in A+ individuals | | |  | Executive function scores in A– individuals | | |
| --- | --- | --- | --- | --- | --- | --- | --- |
|  | β | SE | P |  | β | SE | P |
| Plasma–/CSF– | Reference | Reference | Reference |  | Reference | Reference | Reference |
| Plasma+/CSF– | -0.056 | 0.028 | **0.046** |  | 0.006 | 0.023 | 0.792 |
| Plasma–/CSF+ | -0.119 | 0.029 | **<0.001** |  | -0.013 | 0.022 | 0.552 |
| Plasma+/CSF+ | -0.206 | 0.026 | **<0.001** |  | -0.042 | 0.033 | 0.202 |
|  |  |  |  |  |  |  |  |
| Plasma+/CSF– | Reference | Reference | Reference |  | Reference | Reference | Reference |
| Plasma–/CSF+ | -0.061 | 0.037 | 0.100 |  | -0.020 | 0.032 | 0.532 |
| Plasma+/CSF+ | -0.151 | 0.034 | **<0.001** |  | -0.054 | 0.040 | 0.186 |
|  |  |  |  |  |  |  |  |
| Plasma–/CSF+ | Reference | Reference | Reference |  | Reference | Reference | Reference |
| Plasma+/CSF+ | -0.092 | 0.037 | **0.016** |  | -0.035 | 0.043 | 0.415 |

Survival analyses:


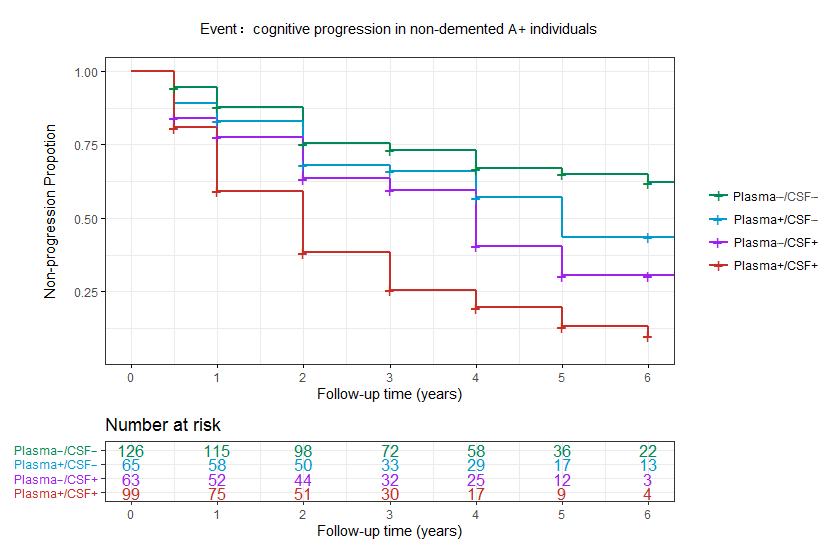


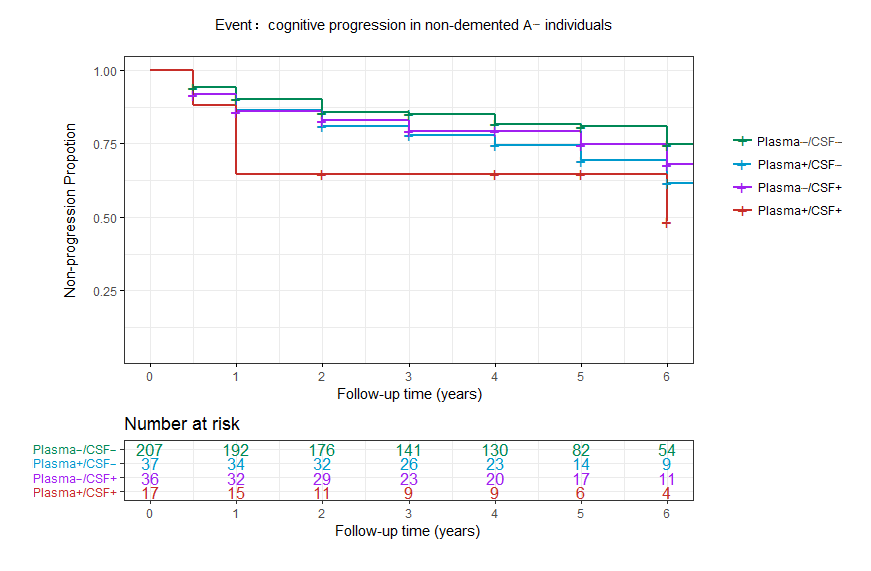


|  | A+ individuals | | | |  | A– individuals | | | |
| --- | --- | --- | --- | --- | --- | --- | --- | --- | --- |
|  | HR | LCI | UCI | P |  | HR | LCI | UCI | P |
| Plasma–/CSF– | Reference | Reference | Reference | Reference |  | Reference | Reference | Reference | Reference |
| Plasma+/CSF– | 1.440 | 0.880 | 2.355 | 0.147 |  | 1.251 | 0.633 | 2.473 | 0.520 |
| Plasma–/CSF+ | 2.085 | 1.306 | 3.330 | **0.002** |  | 1.143 | 0.539 | 2.422 | 0.728 |
| Plasma+/CSF+ | 3.783 | 2.492 | 5.740 | **<0.001** |  | 2.272 | 0.996 | 5.184 | 0.051 |
|  |  |  |  |  |  |  |  |  |  |
| Plasma+/CSF– | Reference | Reference | Reference | Reference |  | Reference | Reference | Reference | Reference |
| Plasma–/CSF+ | 1.450 | 0.880 | 2.390 | 0.145 |  | 0.893 | 0.356 | 2.243 | 0.810 |
| Plasma+/CSF+ | 2.679 | 1.710 | 4.198 | **<0.001** |  | 1.888 | 0.693 | 5.146 | 0.214 |
|  |  |  |  |  |  |  |  |  |  |
| Plasma–/CSF+ | Reference | Reference | Reference | Reference |  | Reference | Reference | Reference | Reference |
| Plasma+/CSF+ | 1.892 | 1.248 | 2.869 | **0.003** |  | 2.161 | 0.721 | 6.477 | 0.169 |

Plasma/PET group:

Longitudinal analyses:

A+ individuals:


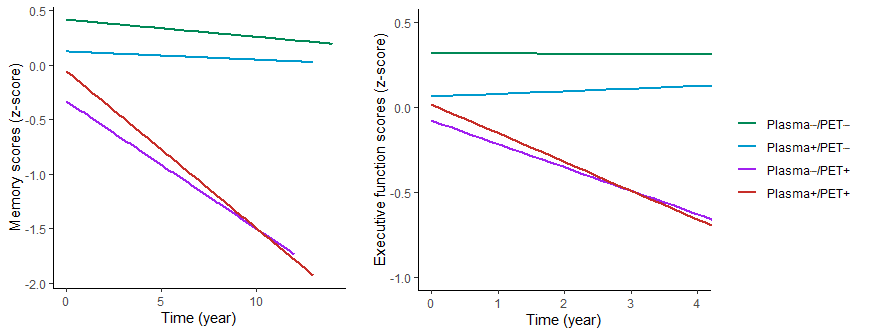


A– individuals:


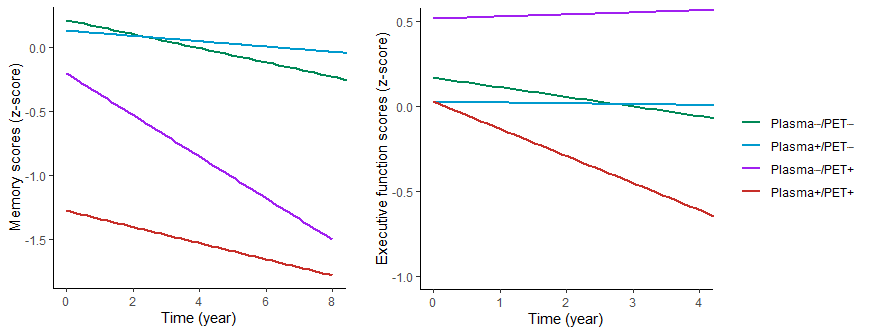


|  | Memory scores in A+ individuals | | |  | Memory scores in A– individuals | | |
| --- | --- | --- | --- | --- | --- | --- | --- |
|  | β | SE | P |  | β | SE | P |
| Plasma–/PET– | Reference | Reference | Reference |  | Reference | Reference | Reference |
| Plasma+/PET– | -0.044 | 0.027 | 0.114 |  | 0.013 | 0.025 | 0.603 |
| Plasma–/PET+ | -0.129 | 0.038 | **0.001** |  | -0.100 | 0.056 | 0.075 |
| Plasma+/PET+ | -0.147 | 0.029 | **<0.001** |  | -0.026 | 0.093 | 0.779 |
|  |  |  |  |  |  |  |  |
| Plasma+/PET– | Reference | Reference | Reference |  | Reference | Reference | Reference |
| Plasma–/PET+ | -0.085 | 0.044 | 0.061 |  | -0.107 | 0.054 | 0.062 |
| Plasma+/PET+ | -0.098 | 0.036 | **0.008** |  | -0.036 | 0.086 | 0.680 |
|  |  |  |  |  |  |  |  |
| Plasma–/PET+ | Reference | Reference | Reference |  | Reference | Reference | Reference |
| Plasma+/PET+ | -0.009 | 0.052 | 0.857 |  | 0.061 | 0.181 | 0.771 |

|  | Executive function scores in A+ individuals | | |  | Executive function scores in A– individuals | | |
| --- | --- | --- | --- | --- | --- | --- | --- |
|  | β | SE | P |  | β | SE | P |
| Plasma–/PET– | Reference | Reference | Reference |  | Reference | Reference | Reference |
| Plasma+/PET– | -0.052 | 0.028 | 0.067 |  | 0.001 | 0.025 | 0.982 |
| Plasma–/PET+ | -0.140 | 0.038 | **0.001** |  | 0.038 | 0.055 | 0.494 |
| Plasma+/PET+ | -0.203 | 0.030 | **<0.001** |  | -0.133 | 0.089 | 0.141 |
|  |  |  |  |  |  |  |  |
| Plasma+/PET– | Reference | Reference | Reference |  | Reference | Reference | Reference |
| Plasma–/PET+ | -0.082 | 0.047 | 0.089 |  | 0.044 | 0.061 | 0.480 |
| Plasma+/PET+ | -0.143 | 0.038 | **<0.001** |  | -0.152 | 0.094 | 0.130 |
|  |  |  |  |  |  |  |  |
| Plasma–/PET+ | Reference | Reference | Reference |  | Reference | Reference | Reference |
| Plasma+/PET+ | -0.063 | 0.061 | 0.306 |  | -0.218 | 0.166 | 0.327 |

Survival analyses:


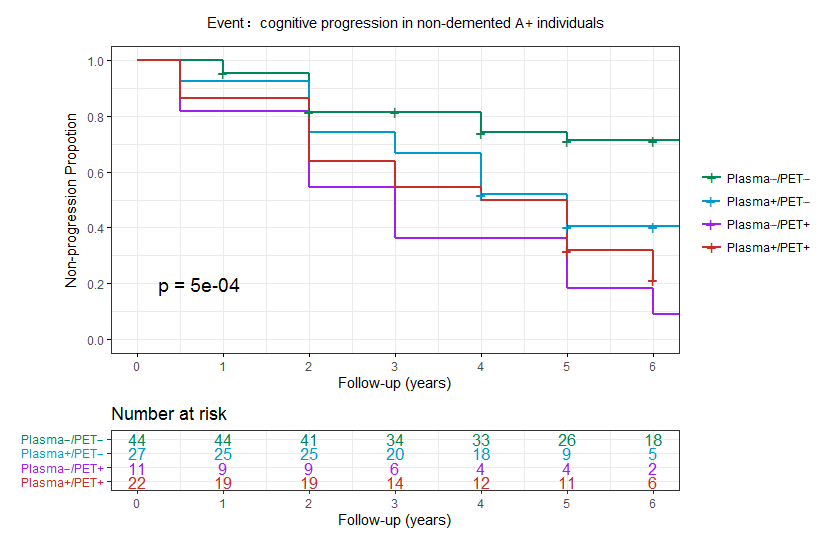


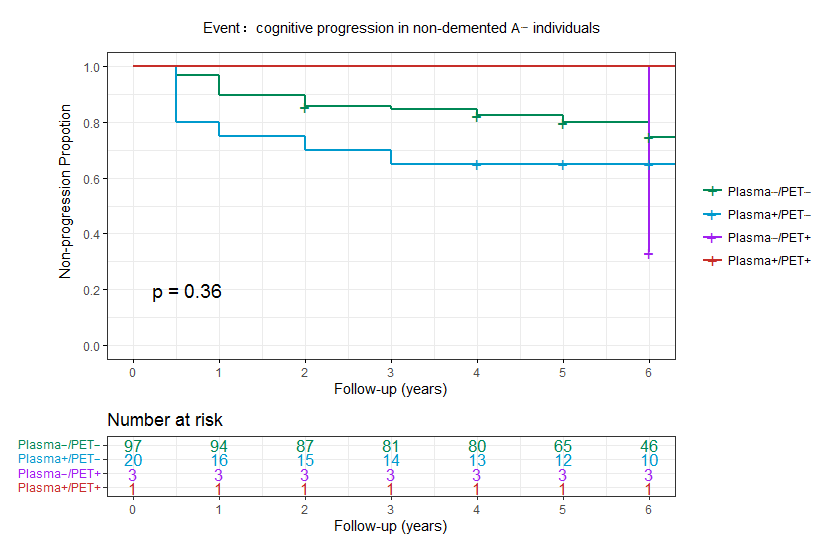


|  | A+ individuals | | | |  | A– individuals | | | |
| --- | --- | --- | --- | --- | --- | --- | --- | --- | --- |
|  | HR | LCI | UCI | P |  | HR | LCI | UCI | P |
| Plasma–/PET– | Reference | Reference | Reference | Reference |  | Reference | Reference | Reference | Reference |
| Plasma+/PET– | 2.155 | 0.990 | 4.688 | 0.053 |  | 1.716 | 0.719 | 4.093 | 0.224 |
| Plasma–/PET+ | 4.199 | 1.744 | 10.108 | **0.001** |  | - | - | - | - |
| Plasma+/PET+ | 2.617 | 1.250 | 5.478 | **0.011** |  | - | - | - | - |
|  |  |  |  |  |  |  |  |  |  |
| Plasma+/PET– | Reference | Reference | Reference | Reference |  | Reference | Reference | Reference | Reference |
| Plasma–/PET+ | 1.832 | 0.760 | 4.419 | 0.178 |  | - | - | - | - |
| Plasma+/PET+ | 1.058 | 0.499 | 2.242 | 0.883 |  | - | - | - | - |
|  |  |  |  |  |  |  |  |  |  |
| Plasma–/PET+ | Reference | Reference | Reference | Reference |  | Reference | Reference | Reference | Reference |
| Plasma+/PET+ | 0.507 | 0.196 | 1.309 | 0.161 |  | - | - | - | - |

Abbreviations: Aβ = amyloid-β, CSF = cerebrospinal fluid, HR = hazard ratio, LCI = lower confidence interval, PET = positron emission tomography, SE = standard error, UCI = upper confidence interval.

Appendix 9: Results after removing borderline cases

Plasma/CSF group:

Cross-sectional analyses:

**
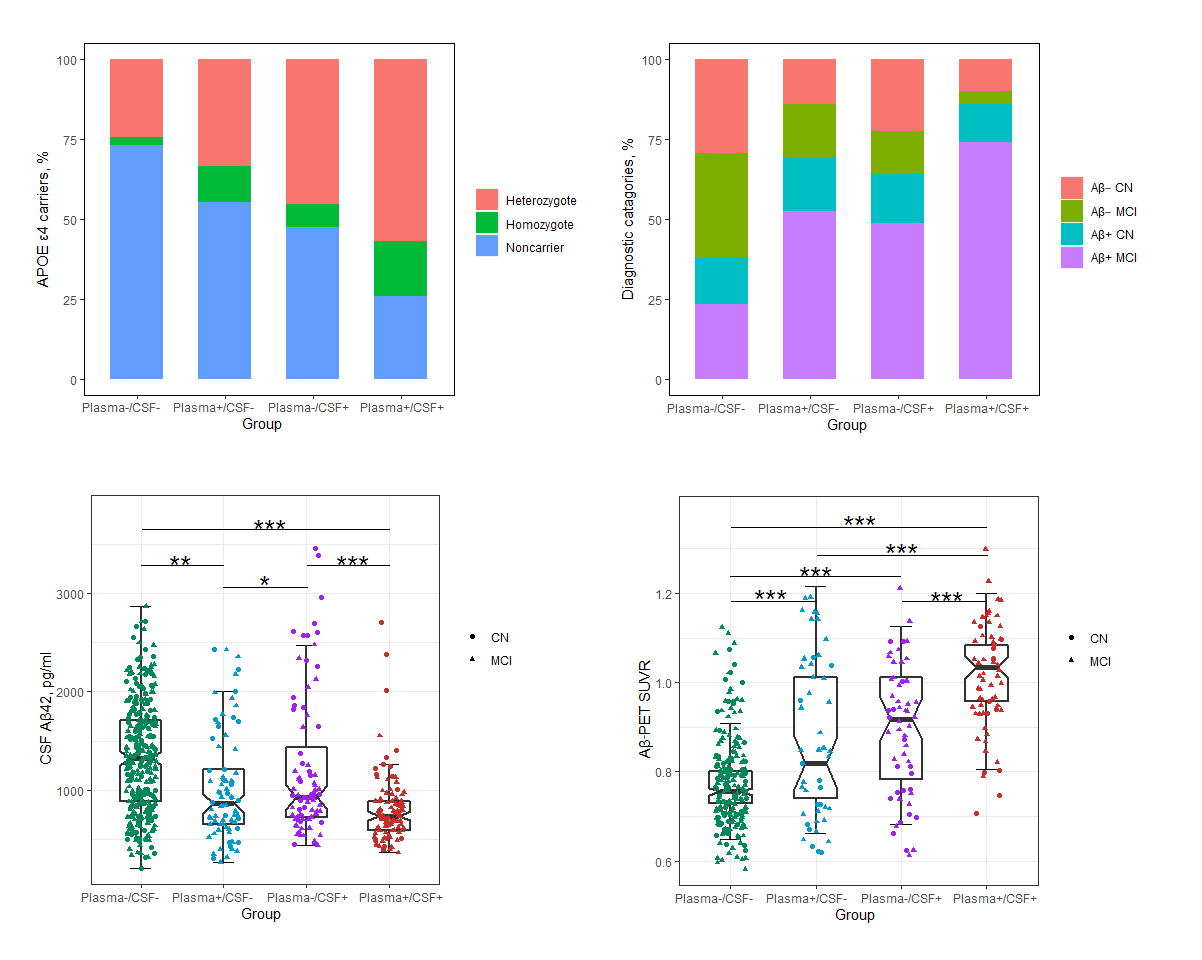
**

**Note:** * represents p-value < 0.05, ** represents p-value < 0.01, and *** represents p-value < 0.001.

Longitudinal analyses:


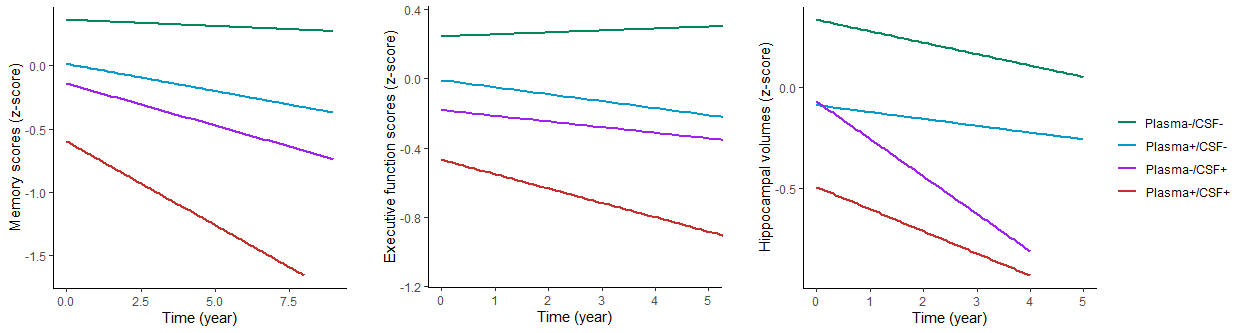


|  | Memory scores | | |  | Executive function scores | | |  | Hippocampal volumes | | |
| --- | --- | --- | --- | --- | --- | --- | --- | --- | --- | --- | --- |
|  | β | SE | P |  | β | SE | P |  | β | SE | P |
| Plasma–/CSF– | Reference | Reference | Reference |  | Reference | Reference | Reference |  | Reference | Reference | Reference |
| Plasma+/CSF– | -0.049 | 0.017 | **0.005** |  | -0.034 | 0.019 | 0.071 |  | -0.040 | 0.019 | **0.031** |
| Plasma–/CSF+ | -0.075 | 0.017 | **<0.001** |  | -0.076 | 0.018 | **<0.001** |  | -0.090 | 0.018 | **<0.001** |
| Plasma+/CSF+ | -0.185 | 0.017 | **<0.001** |  | -0.186 | 0.019 | **<0.001** |  | -0.106 | 0.017 | **<0.001** |
|  |  |  |  |  |  |  |  |  |  |  |  |
| Plasma+/CSF– | Reference | Reference | Reference |  | Reference | Reference | Reference |  | Reference | Reference | Reference |
| Plasma–/CSF+ | -0.024 | 0.025 | 0.338 |  | -0.043 | 0.029 | 0.142 |  | -0.053 | 0.029 | 0.065 |
| Plasma+/CSF+ | -0.137 | 0.025 | **<0.001** |  | -0.156 | 0.029 | **<0.001** |  | -0.071 | 0.028 | **0.010** |
|  |  |  |  |  |  |  |  |  |  |  |  |
| Plasma–/CSF+ | Reference | Reference | Reference |  | Reference | Reference | Reference |  | Reference | Reference | Reference |
| Plasma+/CSF+ | -0.113 | 0.027 | **<0.001** |  | -0.115 | 0.030 | **<0.001** |  | -0.017 | 0.025 | 0.503 |

Survival analyses:

**
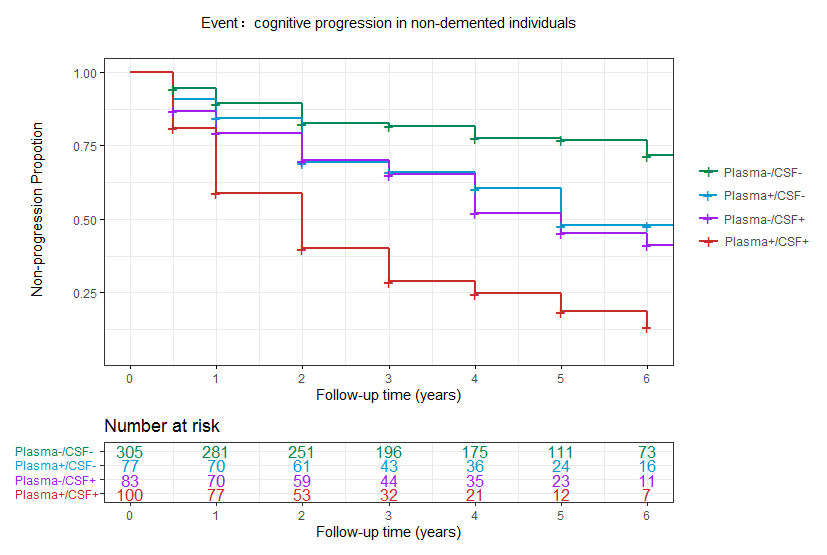
**

|  | HR | LCI | UCI | P |
| --- | --- | --- | --- | --- |
| Plasma–/CSF– | Reference | Reference | Reference | Reference |
| Plasma+/CSF– | 1.814 | 1.181 | 2.786 | **0.007** |
| Plasma–/CSF+ | 2.091 | 1.381 | 3.166 | **<0.001** |
| Plasma+/CSF+ | 4.125 | 2.827 | 6.017 | **<0.001** |
|  |  |  |  |  |
| Plasma+/CSF– | Reference | Reference | Reference | Reference |
| Plasma–/CSF+ | 1.119 | 0.694 | 1.804 | 0.644 |
| Plasma+/CSF+ | 2.262 | 1.461 | 3.504 | **<0.001** |
|  |  |  |  |  |
| Plasma–/CSF+ | Reference | Reference | Reference | Reference |
| Plasma+/CSF+ | 2.045 | 1.361 | 3.075 | **0.001** |

Plasma/PET group:

Cross-sectional analyses:

**
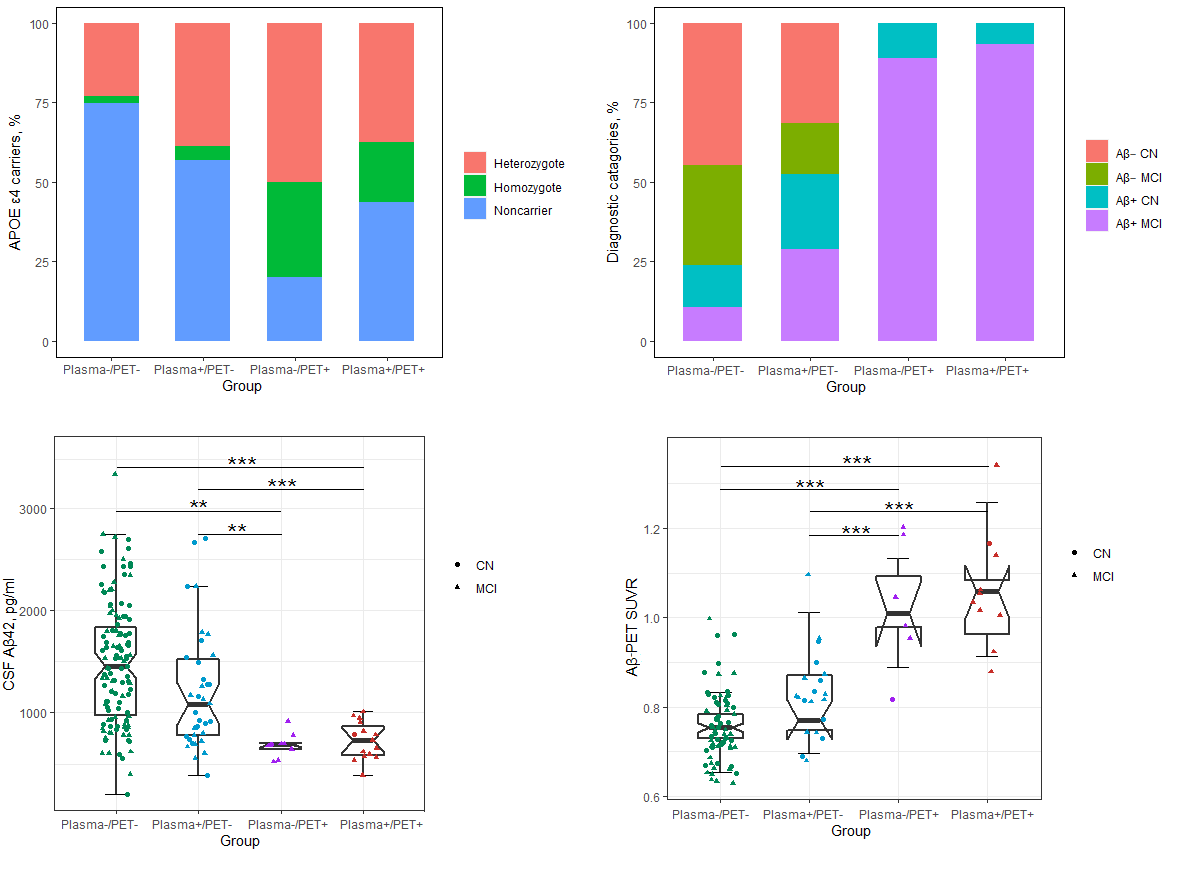
**

**Note:** * represents p-value < 0.05, ** represents p-value < 0.01, and *** represents p-value < 0.001.

Longitudinal analyses:

**
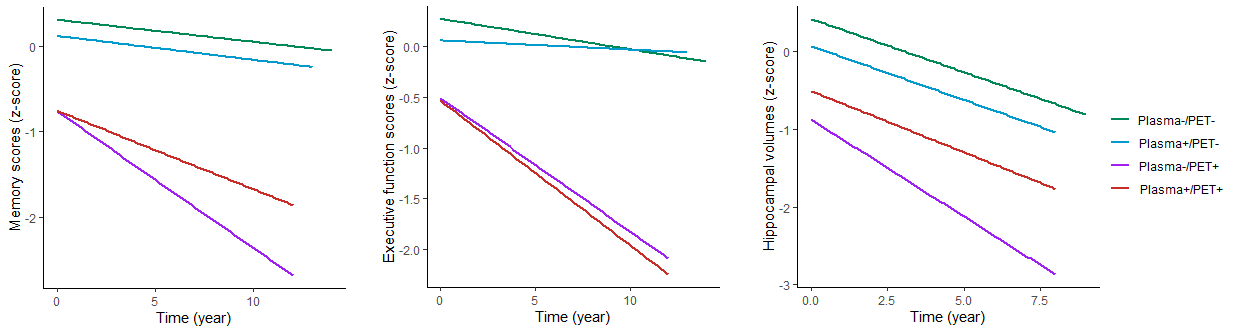
**

|  | Memory scores | | |  | Executive function scores | | |  | Hippocampal volumes | | |
| --- | --- | --- | --- | --- | --- | --- | --- | --- | --- | --- | --- |
|  | β | SE | P |  | β | SE | P |  | β | SE | P |
| Plasma–/PET– | Reference | Reference | Reference |  | Reference | Reference | Reference |  | Reference | Reference | Reference |
| Plasma+/PET– | -0.012 | 0.016 | 0.459 |  | -0.019 | 0.017 | 0.264 |  | 0.008 | 0.022 | 0.707 |
| Plasma–/PET+ | -0.148 | 0.031 | **<0.001** |  | -0.126 | 0.032 | **<0.001** |  | -0.090 | 0.041 | **0.029** |
| Plasma+/PET+ | -0.171 | 0.025 | **<0.001** |  | -0.200 | 0.027 | **<0.001** |  | -0.138 | 0.034 | **<0.001** |
|  |  |  |  |  |  |  |  |  |  |  |  |
| Plasma+/PET– | Reference | Reference | Reference |  | Reference | Reference | Reference |  | Reference | Reference | Reference |
| Plasma–/PET+ | -0.132 | 0.037 | **0.001** |  | -0.108 | 0.042 | **0.012** |  | -0.098 | 0.053 | 0.068 |
| Plasma+/PET+ | -0.149 | 0.031 | **<0.001** |  | -0.189 | 0.035 | **<0.001** |  | -0.154 | 0.045 | **0.001** |
|  |  |  |  |  |  |  |  |  |  |  |  |
| Plasma–/PET+ | Reference | Reference | Reference |  | Reference | Reference | Reference |  | Reference | Reference | Reference |
| Plasma+/PET+ | -0.012 | 0.067 | 0.855 |  | -0.101 | 0.079 | 0.216 |  | -0.058 | 0.061 | 0.354 |

Survival analyses:

**
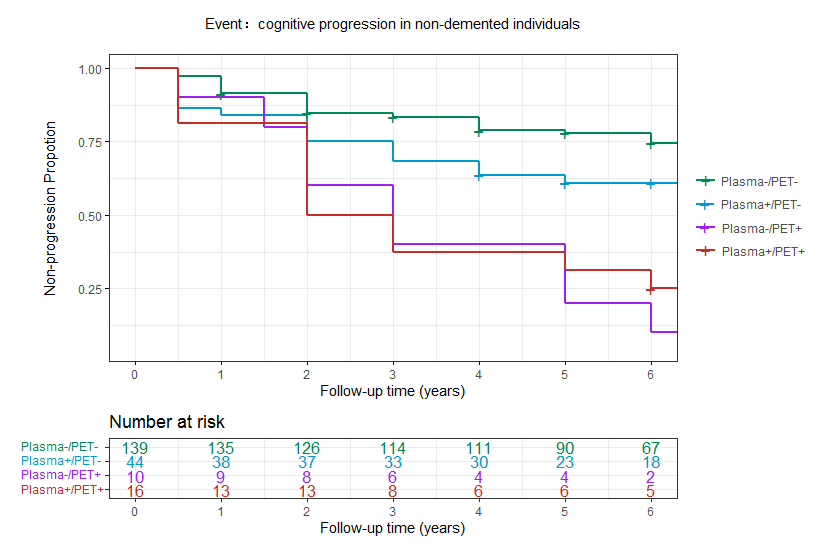
**

|  | HR | LCI | UCI | P |
| --- | --- | --- | --- | --- |
| Plasma–/PET– | Reference | Reference | Reference | Reference |
| Plasma+/PET– | 1.827 | 1.036 | 3.222 | **0.037** |
| Plasma–/PET+ | 5.618 | 2.397 | 13.167 | **<0.001** |
| Plasma+/PET+ | 3.909 | 1.921 | 7.955 | **<0.001** |
|  |  |  |  |  |
| Plasma+/PET– | Reference | Reference | Reference | Reference |
| Plasma–/PET+ | 3.324 | 1.386 | 7.971 | **0.007** |
| Plasma+/PET+ | 2.873 | 1.300 | 6.350 | **0.009** |
|  |  |  |  |  |
| Plasma–/PET+ | Reference | Reference | Reference | Reference |
| Plasma+/PET+ | 0.954 | 0.341 | 2.675 | 0.929 |

Abbreviations: Aβ = amyloid-β, CN = cognitively normal, CSF = cerebrospinal fluid, HR = hazard ratio, LCI = lower confidence interval, MCI = mild cognitive impairment, PET = positron emission tomography, SE = standard error, SUVR = standard uptake value ratio, UCI = upper confidence interval.

Appendix 10: Results using concurrent tau measures

Plasma/PET group (participants had plasma p-tau181 and tau-PET assessments at the same visit**):**

Sample characteristics:

| Characteristics | Plasma/PET group | | | | |
| --- | --- | --- | --- | --- | --- |
|  | Plasma–/PET– | Plasma+/PET– | Plasma–/PET+ | Plasma+/PET+ | P |
| Numbers | 27 | 9 | 4 | 4 |  |
| Age (years) | 70.15 (6.21) | 72.78 (8.16) | 72.08 (5.24) | 71.53 (4.06) | 0.733 |
| Female (%) | 11 (40.7) | 4 (44.4) | 3 (75.0) | 3 (75.0) | 0.399 |
| Educational years | 17.15 (2.32) | 16.78 (2.68) | 17.00 (1.15) | 16.50 (3.42) | 0.951 |
| *APOE* Ɛ4 (%) | 7 (25.9) | 5 (55.6) | 2 (50.0) | 1 (25.0) | 0.355 |
| Diagnosis |  |  |  |  |  |
| CN | 14 (51.9) | 5 (55.6) | 2 (50.0) | 2 (50.0) | 0.996 |
| MCI | 13 (48.1) | 4 (44.4) | 2 (50.0) | 2 (50.0) |  |

Note: Continuous variables were presented as means (standard deviations (SDs)), and categorical variables were presented as numbers (percents).

Cross-sectional analyses:

**
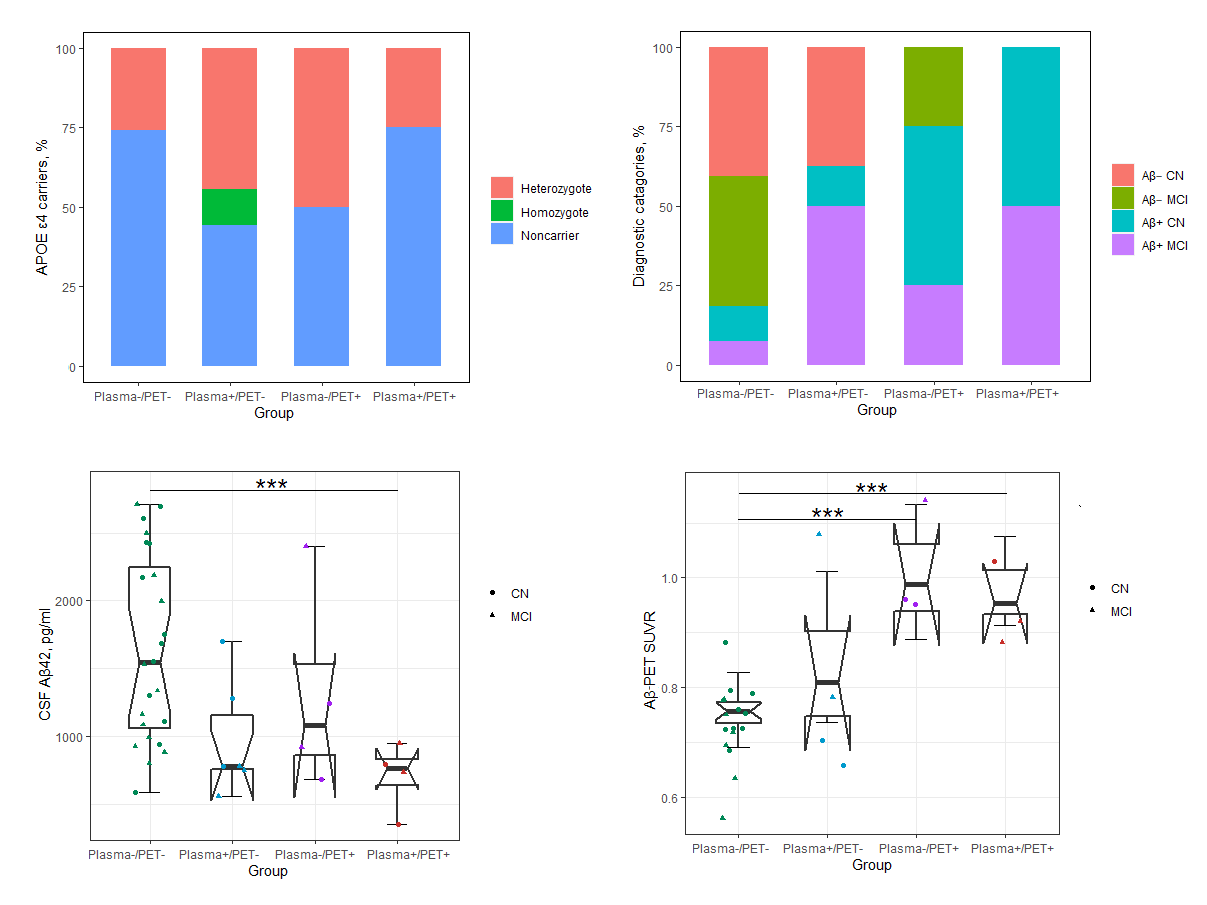
**

**Note:** * represents p-value < 0.05, ** represents p-value < 0.01, and *** represents p-value < 0.001.

Longitudinal analyses:

**
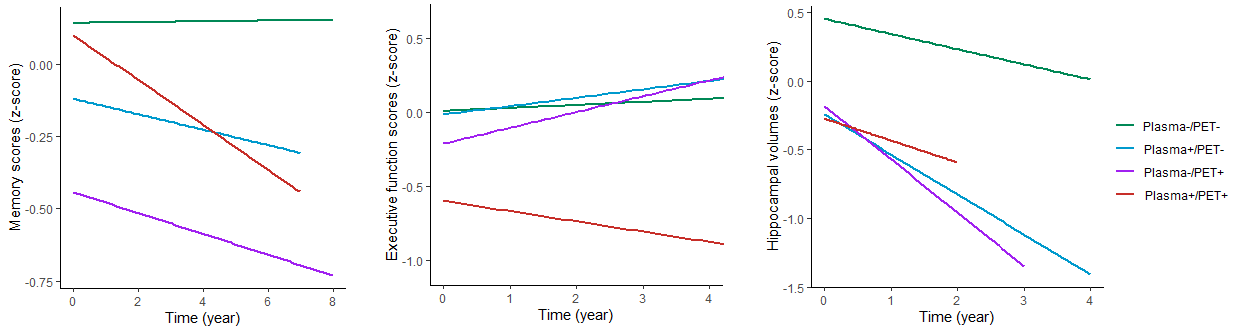
**

Note: We showed the longitudinal trajectories even though the sample size of plasma/PET categories was small.

|  | Memory scores | | |  | Executive function scores | | |  | Hippocampal volumes | | |
| --- | --- | --- | --- | --- | --- | --- | --- | --- | --- | --- | --- |
|  | β | SE | P |  | β | SE | P |  | β | SE | P |
| Plasma–/PET– | Reference | Reference | Reference |  | Reference | Reference | Reference |  | Reference | Reference | Reference |
| Plasma+/PET– | -0.095 | 0.039 | **0.022** |  | -0.044 | 0.053 | 0.416 |  | -0.103 | 0.063 | 0.111 |
| Plasma–/PET+ | -0.148 | 0.052 | **0.009** |  | 0.009 | 0.071 | 0.903 |  | -0.089 | 0.091 | 0.335 |
| Plasma+/PET+ | -0.195 | 0.052 | **0.001** |  | -0.201 | 0.072 | **0.009** |  | -0.071 | 0.088 | 0.425 |
|  |  |  |  |  |  |  |  |  |  |  |  |
| Plasma+/PET– | Reference | Reference | Reference |  | Reference | Reference | Reference |  | Reference | Reference | Reference |
| Plasma–/PET+ | -0.062 | 0.080 | 0.457 |  | 0.042 | 0.099 | 0.683 |  | 0.027 | 0.140 | 0.852 |
| Plasma+/PET+ | -0.084 | 0.080 | 0.316 |  | -0.147 | 0.100 | 0.165 |  | 0.028 | 0.136 | 0.841 |
|  |  |  |  |  |  |  |  |  |  |  |  |
| Plasma–/PET+ | Reference | Reference | Reference |  | Reference | Reference | Reference |  | Reference | Reference | Reference |
| Plasma+/PET+ | -0.011 | 0.125 | 0.931 |  | -0.178 | 0.110 | 0.161 |  | 0.008 | 0.172 | 0.966 |

Survival analyses:

**
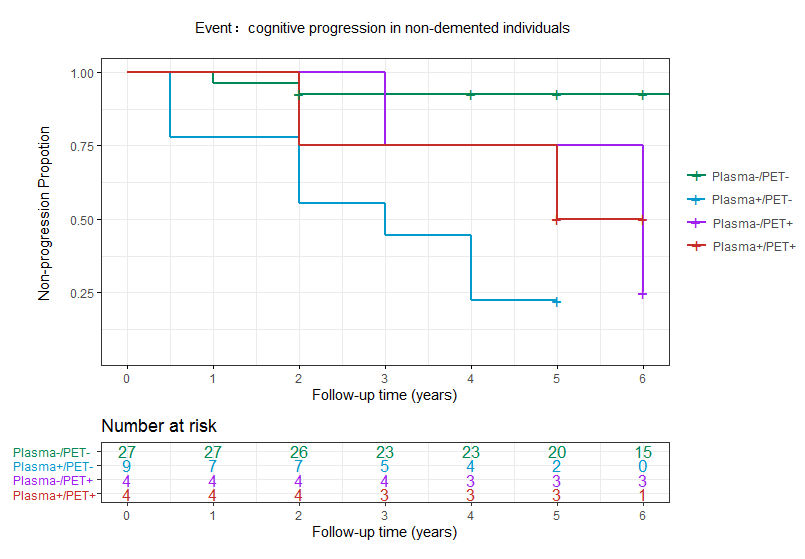
**

|  | HR | LCI | UCI | P |
| --- | --- | --- | --- | --- |
| Plasma–/PET– | Reference | Reference | Reference | Reference |
| Plasma+/PET– | 31.407 | 5.015 | 196.695 | **<0.001** |
| Plasma–/PET+ | 9.689 | 1.258 | 74.603 | **0.029** |
| Plasma+/PET+ | 8.814 | 1.116 | 69.623 | **0.039** |
|  |  |  |  |  |
| Plasma+/PET– | Reference | Reference | Reference | Reference |
| Plasma–/PET+ | 0.158 | 0.017 | 1.474 | 0.105 |
| Plasma+/PET+ | 0.228 | 0.037 | 1.427 | 0.114 |
|  |  |  |  |  |
| Plasma–/PET+ | Reference | Reference | Reference | Reference |
| Plasma+/PET+ | - | - | - | - |

Abbreviations: Aβ = amyloid-β, CN = cognitively normal, CSF = cerebrospinal fluid, HR = hazard ratio, LCI = lower confidence interval, MCI = mild cognitive impairment, PET = positron emission tomography, SE = standard error, SUVR = standard uptake value ratio, UCI = upper confidence interval.

Appendix 11: Results using plasma p-tau181 and tau-PET assessments within a 12-month interval

Plasma/PET group**:**

Sample characteristics:

| Characteristics | Plasma/PET group | | | | |
| --- | --- | --- | --- | --- | --- |
|  | Plasma–/PET– | Plasma+/PET– | Plasma–/PET+ | Plasma+/PET+ | P |
| Numbers (%) | 61 (62.9) | 22 (22.7) | 5 (5.2) | 9 (9.3) |  |
| Age (years) | 70.08 (6.59) | 73.98 (6.95) | 73.08 (5.06) | 70.18 (7.64) | 0.116 |
| Female (%) | 25 (41.0) | 6 (27.3) | 3 (60.0) | 7 (77.8) | 0.061 |
| Educational years | 16.44 (2.55) | 16.59 (2.77) | 16.00 (2.45) | 15.33 (3.32) | 0.651 |
| *APOE* Ɛ4 (%) | 21 (34.4) | 11 (50.0) | 3 (60.0) | 4 (44.4) | 0.456 |
| Diagnosis | 6.69 (6.01) | 7.09 (6.04) | 2.40 (5.37) | 6.67 (6.32) | 0.463 |
| CN | 26 (42.6) | 7 (31.8) | 2 (40.0) | 3 (33.3) | 0.817 |
| MCI | 35 (57.4) | 15 (68.2) | 3 (60.0) | 6 (66.7) |  |

Note: Continuous variables were presented as means (standard deviations (SDs)), and categorical variables were presented as numbers (percents).

Cross-sectional analyses:


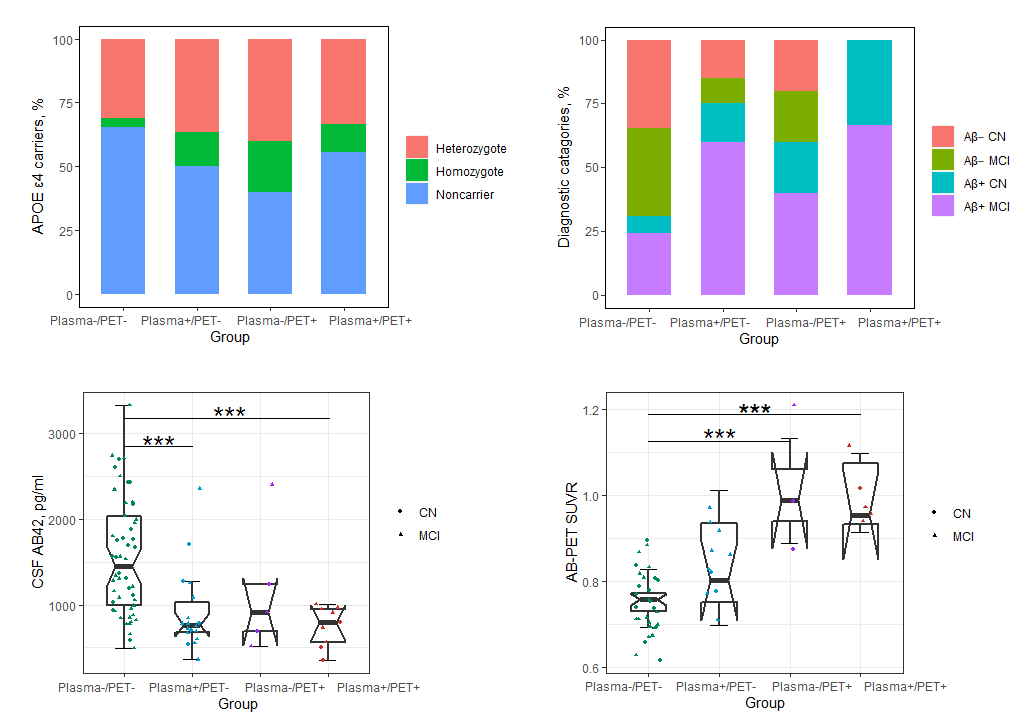


Longitudinal analyses:


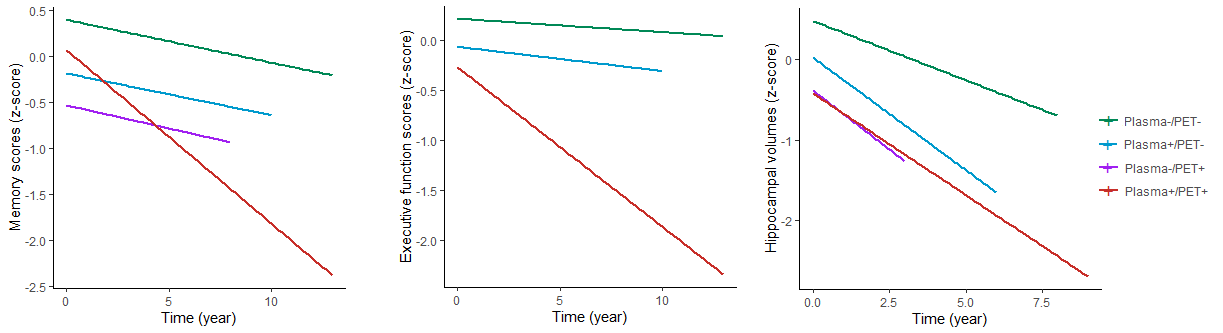


Note: Since only 5 plasma–/PET+ persons could be included in the longitudinal analyses of executive function scores, their relevant trajectories were not shown.

|  | Memory scores | | |  | Executive function scores | | |  | Hippocampal volumes | | |
| --- | --- | --- | --- | --- | --- | --- | --- | --- | --- | --- | --- |
|  | β | SE | P |  | β | SE | P |  | β | SE | P |
| Plasma–/PET– | Reference | Reference | Reference |  | Reference | Reference | Reference |  | Reference | Reference | Reference |
| Plasma+/PET– | -0.039 | 0.028 | 0.175 |  | -0.035 | 0.032 | 0.284 |  | -0.045 | 0.038 | 0.234 |
| Plasma–/PET+ | -0.115 | 0.053 | **0.032** |  | -0.007 | 0.060 | 0.909 |  | -0.051 | 0.073 | 0.486 |
| Plasma+/PET+ | -0.248 | 0.040 | **<0.001** |  | -0.280 | 0.045 | **<0.001** |  | -0.125 | 0.050 | **0.015** |
|  |  |  |  |  |  |  |  |  |  |  |  |
| Plasma+/PET– | Reference | Reference | Reference |  | Reference | Reference | Reference |  | Reference | Reference | Reference |
| Plasma–/PET+ | -0.076 | 0.063 | 0.242 |  | 0.023 | 0.073 | 0.753 |  | 0.005 | 0.103 | 0.963 |
| Plasma+/PET+ | -0.191 | 0.050 | **0.001** |  | -0.221 | 0.058 | **0.001** |  | -0.094 | 0.077 | 0.229 |
|  |  |  |  |  |  |  |  |  |  |  |  |
| Plasma–/PET+ | Reference | Reference | Reference |  | Reference | Reference | Reference |  | Reference | Reference | Reference |
| Plasma+/PET+ | 0.742 | 0.602 | 0.257 |  | -0.243 | 0.106 | 0.042 |  | -0.101 | 0.115 | 0.395 |

Survival analyses:


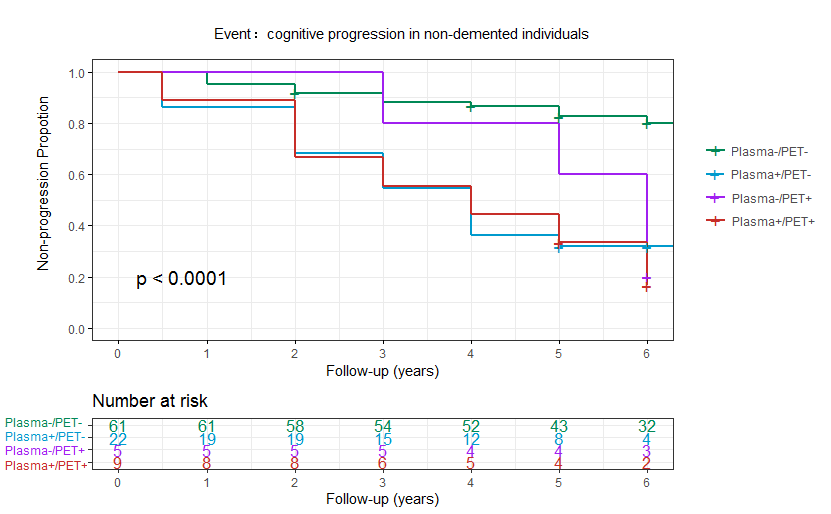


|  | HR | LCI | UCI | P |
| --- | --- | --- | --- | --- |
| Plasma–/PET– | Reference | Reference | Reference | Reference |
| Plasma+/PET– | 5.759 | 2.579 | 12.859 | **<0.001** |
| Plasma–/PET+ | 4.418 | 1.278 | 15.273 | **0.019** |
| Plasma+/PET+ | 5.764 | 2.098 | 15.834 | **0.001** |
|  |  |  |  |  |
| Plasma+/PET– | Reference | Reference | Reference | Reference |
| Plasma–/PET+ | 0.522 | 0.140 | 1.953 | 0.334 |
| Plasma+/PET+ | 0.915 | 0.324 | 2.582 | 0.866 |
|  |  |  |  |  |
| Plasma–/PET+ | Reference | Reference | Reference | Reference |
| Plasma+/PET+ | 0.972 | 0.168 | 5.640 | 0.975 |

Abbreviations: Aβ = amyloid-β, CN = cognitively normal, CSF = cerebrospinal fluid, HR = hazard ratio, LCI = lower confidence interval, MCI = mild cognitive impairment, PET = positron emission tomography, SE = standard error, SUVR = standard uptake value ratio, UCI = upper confidence interval.

Appendix 12: Results using an alternative ROI (entorhinal cortex)

Plasma/PET group**:**

Sample characteristics:

| Characteristics | Plasma/PET group | | | | |
| --- | --- | --- | --- | --- | --- |
|  | Plasma–/PET– | Plasma+/PET– | Plasma–/PET+ | Plasma+/PET+ | P |
| Numbers (%) | 156 (63.7) | 59 (24.1) | 10 (4.1) | 20 (8.2) |  |
| Age (years) | 70.57 (6.38) | 74.09 (7.12) | 72.22 (6.55) | 71.37 (7.15) | **0.008** |
| Female (%) | 77 (49.4) | 22 (37.3) | 4 (40.0) | 14 (70.0) | 0.073 |
| Educational years | 16.40 (2.62) | 16.58 (2.65) | 15.40 (3.57) | 14.90 (2.63) | 0.060 |
| *APOE* Ɛ4 (%) | 45 (28.8) | 24 (40.7) | 7 (70.0) | 11 (55.0) | **0.007** |
| Diagnosis |  |  |  |  |  |
| CN | 85 (54.5) | 33 (55.9) | 1 (10.0) | 3 (15.0) | **<0.001** |
| MCI | 71 (45.5) | 26 (44.1) | 9 (90.0) | 17 (85.0) |  |

Note: Continuous variables were presented as means (standard deviations (SDs)), and categorical variables were presented as numbers (percents).

Cross-sectional analyses:


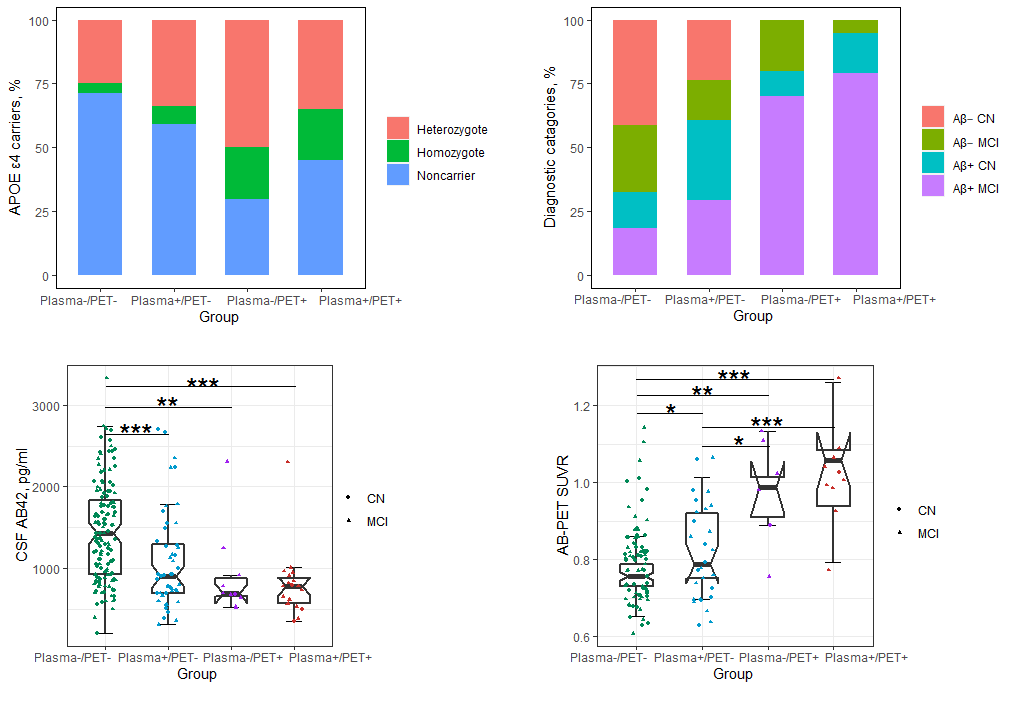


Longitudinal analyses:


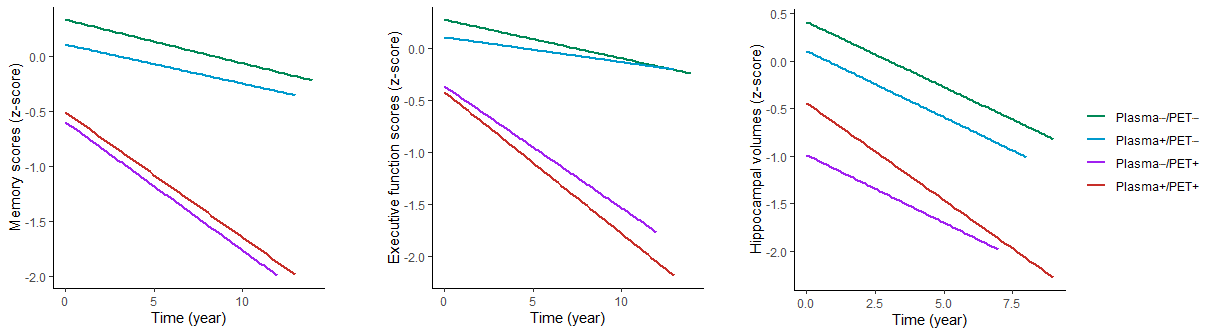


|  | Memory scores | | |  | Executive function scores | | |  | Hippocampal volumes | | |
| --- | --- | --- | --- | --- | --- | --- | --- | --- | --- | --- | --- |
|  | β | SE | P |  | β | SE | P |  | β | SE | P |
| Plasma–/PET– | Reference | Reference | Reference |  | Reference | Reference | Reference |  | Reference | Reference | Reference |
| Plasma+/PET– | -0.021 | 0.015 | 0.163 |  | -0.032 | 0.015 | **0.042** |  | -0.002 | 0.019 | 0.915 |
| Plasma–/PET+ | -0.136 | 0.032 | **<0.001** |  | -0.102 | 0.032 | **0.002** |  | -0.081 | 0.038 | **0.032** |
| Plasma+/PET+ | -0.152 | 0.024 | **<0.001** |  | -0.186 | 0.025 | **<0.001** |  | -0.130 | 0.028 | **<0.001** |
|  |  |  |  |  |  |  |  |  |  |  |  |
| Plasma+/PET– | Reference | Reference | Reference |  | Reference | Reference | Reference |  | Reference | Reference | Reference |
| Plasma–/PET+ | -0.118 | 0.038 | **0.003** |  | -0.074 | 0.041 | 0.076 |  | -0.080 | 0.047 | 0.093 |
| Plasma+/PET+ | -0.129 | 0.029 | **<0.001** |  | -0.165 | 0.032 | **<0.001** |  | -0.132 | 0.036 | **<0.001** |
|  |  |  |  |  |  |  |  |  |  |  |  |
| Plasma–/PET+ | Reference | Reference | Reference |  | Reference | Reference | Reference |  | Reference | Reference | Reference |
| Plasma+/PET+ | -0.004 | 0.063 | 0.950 |  | -0.103 | 0.070 | 0.153 |  | -0.053 | 0.054 | 0.339 |

Survival analyses:

**
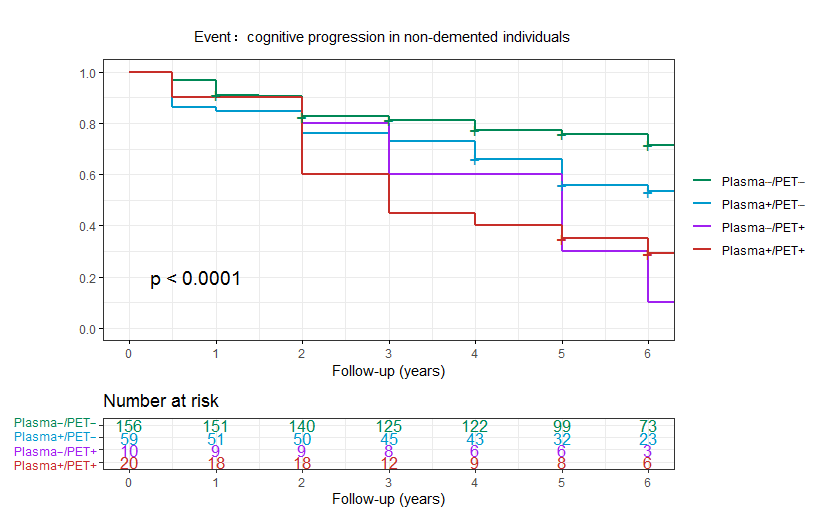
**

|  | HR | LCI | UCI | P |
| --- | --- | --- | --- | --- |
| Plasma–/PET– | Reference | Reference | Reference | Reference |
| Plasma+/PET– | 1.665 | 1.022 | 2.712 | **0.041** |
| Plasma–/PET+ | 3.074 | 1.408 | 6.710 | **0.005** |
| Plasma+/PET+ | 2.538 | 1.350 | 4.772 | **0.004** |
|  |  |  |  |  |
| Plasma+/PET– | Reference | Reference | Reference | Reference |
| Plasma–/PET+ | 2.470 | 1.082 | 5.638 | **0.032** |
| Plasma+/PET+ | 1.762 | 0.876 | 3.545 | 0.112 |
|  |  |  |  |  |
| Plasma–/PET+ | Reference | Reference | Reference | Reference |
| Plasma+/PET+ | 0.734 | 0.287 | 1.879 | 0.519 |

Abbreviations: Aβ = amyloid-β, CN = cognitively normal, CSF = cerebrospinal fluid, HR = hazard ratio, LCI = lower confidence interval, MCI = mild cognitive impairment, PET = positron emission tomography, ROI = regions of interest, SE = standard error, SUVR = standard uptake value ratio, UCI = upper confidence interval.

Note: Tau-PET status was defined based on the SUVR in the entorhinal ROI and using a predefined cutoff of 1.48 SUVR (Reference: Braak H, Braak E. Neuropathological stageing of Alzheimer-related changes. Acta Neuropathol. 1991;82(4):239-259).

Appendix 13: Results using previous cut-off for plasma p-tau181

Plasma/CSF group (plasma p-tau181 concentrations > 17.7 pg/ml were considered positive):

Sample characteristics:

| Characteristics | Plasma/CSF group | | | | |
| --- | --- | --- | --- | --- | --- |
|  | Plasma–/CSF– | Plasma+/CSF– | Plasma–/CSF+ | Plasma+/CSF+ | P |
| Numbers (%) | 331 (49.6) | 118 (17.7) | 91 (13.6) | 128 (19.2) |  |
| Age (years) | 70.38 (6.55) | 72.62 (7.28) | 74.15 (7.00) | 74.07 (6.86) | **<0.001** |
| Female (%) | 161 (48.6) | 48 (40.7) | 47 (51.6) | 66 (51.6) | 0.294 |
| Educational years | 16.42 (2.51) | 16.36 (2.76) | 16.49 (2.60) | 16.19 (2.56) | 0.804 |
| *APOE* Ɛ4 (%) | 89 (26.9) | 51 (43.2) | 49 (53.8) | 84 (65.6) | **<0.001** |
| Diagnosis |  |  |  |  |  |
| CN | 149 (45.0) | 42 (35.6) | 36 (39.6) | 25 (19.5) | **<0.001** |
| MCI | 182 (55.0) | 76 (64.4) | 55 (60.4) | 103 (80.5) |  |

Note: Continuous variables were presented as means (standard deviations (SDs)), and categorical variables were presented as numbers (percents).

Longitudinal analyses:


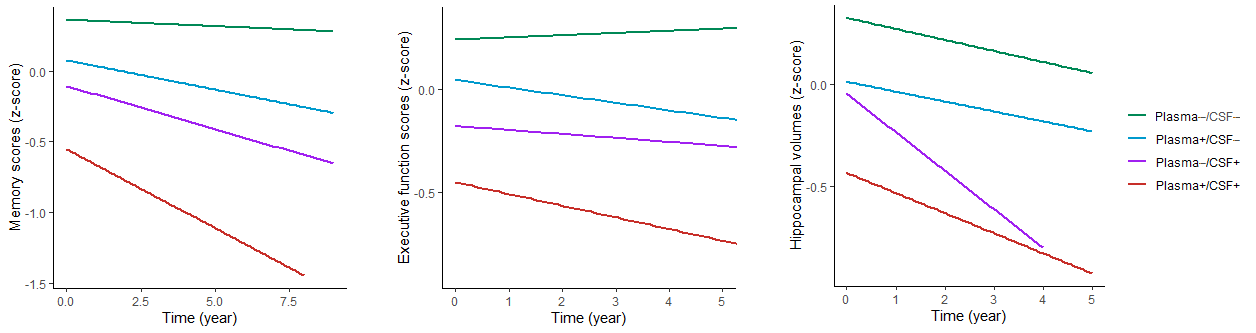


|  | Memory scores | | |  | Executive function scores | | |  | Hippocampal volumes | | |
| --- | --- | --- | --- | --- | --- | --- | --- | --- | --- | --- | --- |
|  | β | SE | P |  | β | SE | P |  | β | SE | P |
| Plasma–/CSF– | Reference | Reference | Reference |  | Reference | Reference | Reference |  | Reference | Reference | Reference |
| Plasma+/CSF– | -0.048 | 0.015 | **0.002** |  | -0.031 | 0.016 | 0.055 |  | -0.034 | 0.016 | **0.033** |
| Plasma–/CSF+ | -0.069 | 0.017 | **<0.001** |  | -0.062 | 0.018 | **0.001** |  | -0.080 | 0.017 | **<0.001** |
| Plasma+/CSF+ | -0.176 | 0.016 | **<0.001** |  | -0.175 | 0.017 | **<0.001** |  | -0.091 | 0.015 | **<0.001** |
|  |  |  |  |  |  |  |  |  |  |  |  |
| Plasma+/CSF– | Reference | Reference | Reference |  | Reference | Reference | Reference |  | Reference | Reference | Reference |
| Plasma–/CSF+ | -0.018 | 0.024 | 0.437 |  | -0.034 | 0.025 | 0.187 |  | -0.048 | 0.024 | 0.050 |
| Plasma+/CSF+ | -0.129 | 0.022 | **<0.001** |  | -0.148 | 0.024 | **<0.001** |  | -0.061 | 0.022 | **0.007** |
|  |  |  |  |  |  |  |  |  |  |  |  |
| Plasma–/CSF+ | Reference | Reference | Reference |  | Reference | Reference | Reference |  | Reference | Reference | Reference |
| Plasma+/CSF+ | -0.111 | 0.027 | **<0.001** |  | -0.114 | 0.028 | **<0.001** |  | -0.012 | 0.023 | 0.588 |

Survival analyses:


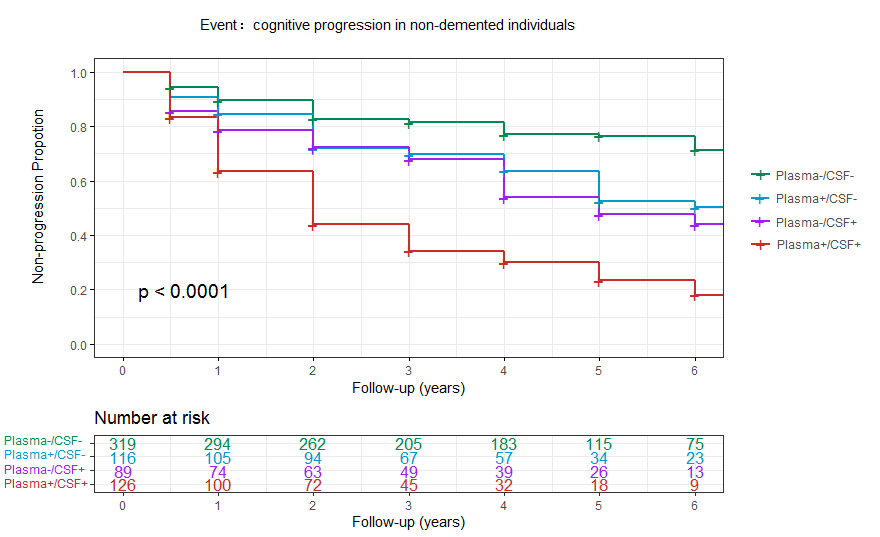


|  | HR | LCI | UCI | P |
| --- | --- | --- | --- | --- |
| Plasma–/CSF– | Reference | Reference | Reference | Reference |
| Plasma+/CSF– | 1.662 | 1.142 | 2.417 | **0.008** |
| Plasma–/CSF+ | 1.947 | 1.305 | 2.905 | **0.001** |
| Plasma+/CSF+ | 3.646 | 2.585 | 5.144 | **<0.001** |
|  |  |  |  |  |
| Plasma+/CSF– | Reference | Reference | Reference | Reference |
| Plasma–/CSF+ | 1.154 | 0.750 | 1.774 | 0.515 |
| Plasma+/CSF+ | 2.193 | 1.511 | 3.183 | **<0.001** |
|  |  |  |  |  |
| Plasma–/CSF+ | Reference | Reference | Reference | Reference |
| Plasma+/CSF+ | 1.913 | 1.299 | 2.816 | **0.001** |

Plasma/PET group (plasma p-tau181 concentrations > 17.7 pg/ml were considered positive):

Sample characteristics:

| Characteristics | Plasma/PET group | | | | |
| --- | --- | --- | --- | --- | --- |
|  | Plasma–/PET– | Plasma+/PET– | Plasma–/PET+ | Plasma+/PET+ | P |
| Numbers (%) | 142 (58.0) | 64 (26.1) | 13 (5.30) | 26 (10.6) |  |
| Age (years) | 70.47 (6.40) | 73.04 (7.19) | 73.43 (5.99) | 72.84 (7.18) | **0.033** |
| Female (%) | 70 (49.3) | 25 (39.1) | 5 (38.5) | 17 (65.4) | 0.124 |
| Educational years | 16.28 (2.74) | 16.56 (2.64) | 16.31 (2.18) | 15.58 (2.79) | 0.482 |
| *APOE* Ɛ4 (%) | 40 (28.2) | 26 (40.6) | 8 (61.5) | 13 (50.0) | **0.016** |
| Diagnosis |  |  |  |  |  |
| CN | 79 (55.6) | 33 (51.6) | 2 (15.4) | 8 (30.8) | **0.008** |
| MCI | 63 (44.4) | 31 (48.4) | 11 (84.6) | 18 (69.2) |  |

Note: Continuous variables were presented as means (standard deviations (SDs)), and categorical variables were presented as numbers (percents).

Longitudinal analyses:


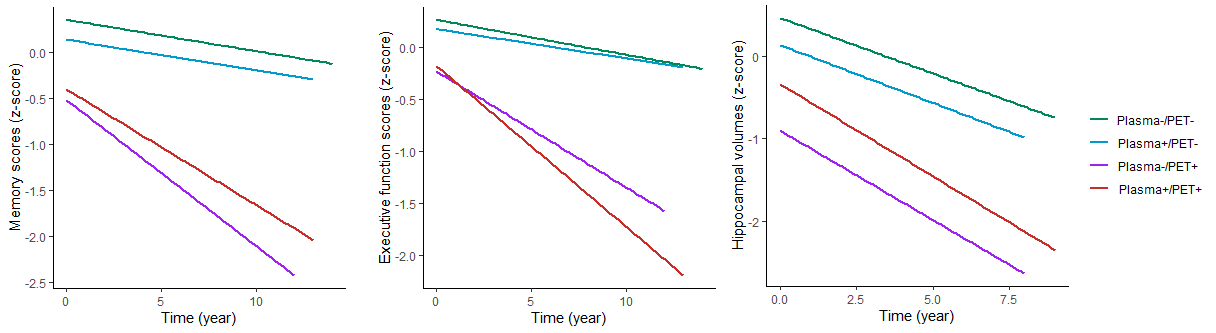


|  | Memory scores | | |  | Executive function scores | | |  | Hippocampal volumes | | |
| --- | --- | --- | --- | --- | --- | --- | --- | --- | --- | --- | --- |
|  | β | SE | P |  | β | SE | P |  | β | SE | P |
| Plasma–/PET– | Reference | Reference | Reference |  | Reference | Reference | Reference |  | Reference | Reference | Reference |
| Plasma+/PET– | -0.020 | 0.015 | 0.183 |  | -0.025 | 0.015 | 0.096 |  | 0.009 | 0.019 | 0.648 |
| Plasma–/PET+ | -0.131 | 0.028 | **<0.001** |  | -0.089 | 0.029 | **0.002** |  | -0.074 | 0.035 | **0.037** |
| Plasma+/PET+ | -0.137 | 0.021 | **<0.001** |  | -0.165 | 0.022 | **<0.001** |  | -0.108 | 0.026 | **<0.001** |
|  |  |  |  |  |  |  |  |  |  |  |  |
| Plasma+/PET– | Reference | Reference | Reference |  | Reference | Reference | Reference |  | Reference | Reference | Reference |
| Plasma–/PET+ | -0.108 | 0.032 | **0.001** |  | -0.064 | 0.036 | 0.076 |  | -0.081 | 0.043 | 0.060 |
| Plasma+/PET+ | -0.111 | 0.025 | **<0.001** |  | -0.143 | 0.028 | **<0.001** |  | -0.118 | 0.033 | **<0.001** |
|  |  |  |  |  |  |  |  |  |  |  |  |
| Plasma–/PET+ | Reference | Reference | Reference |  | Reference | Reference | Reference |  | Reference | Reference | Reference |
| Plasma+/PET+ | 0.001 | 0.046 | 0.977 |  | -0.078 | 0.053 | 0.150 |  | -0.037 | 0.046 | 0.424 |

Survival analyses:


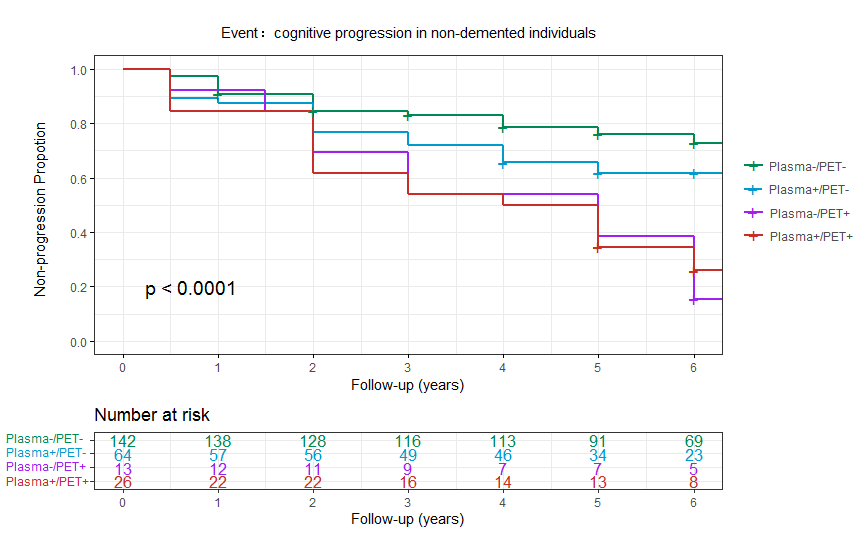


|  | HR | LCI | UCI | P |
| --- | --- | --- | --- | --- |
| Plasma–/PET– | Reference | Reference | Reference | Reference |
| Plasma+/PET– | 1.608 | 0.980 | 2.638 | 0.060 |
| Plasma–/PET+ | 3.652 | 1.795 | 7.427 | **<0.001** |
| Plasma+/PET+ | 2.938 | 1.662 | 5.196 | **<0.001** |
|  |  |  |  |  |
| Plasma+/PET– | Reference | Reference | Reference | Reference |
| Plasma–/PET+ | 1.926 | 0.931 | 3.986 | 0.077 |
| Plasma+/PET+ | 1.915 | 1.040 | 3.525 | **0.037** |
|  |  |  |  |  |
| Plasma–/PET+ | Reference | Reference | Reference | Reference |
| Plasma+/PET+ | 0.803 | 0.356 | 1.810 | 0.597 |

Abbreviations: CN = cognitively normal, CSF = cerebrospinal fluid, HR = hazard ratio, LCI = lower confidence interval, MCI = mild cognitive impairment, PET = positron emission tomography, SE = standard error, UCI = upper confidence interval.
